# Synaptic and network consequences of monosynaptic nociceptive inputs of parabrachial nucleus origin in the central amygdala

**DOI:** 10.1152/jn.00946.2015

**Published:** 2016-02-17

**Authors:** Yae K. Sugimura, Yukari Takahashi, Ayako M. Watabe, Fusao Kato

**Affiliations:** ^1^Department of Neuroscience, The Jikei University School of Medicine, Minato, Tokyo, Japan;; ^2^Center for Neuroscience of Pain, The Jikei University School of Medicine, Minato, Tokyo, Japan;; ^3^Research Fellow of Japan Society for the Promotion of Science, Tokyo, Japan;; ^4^Precursory Research for Embryonic Science and Technology, Japan Science and Technology Agency, Kawaguchi, Saitama, Japan; and; ^5^Nagoya University Graduate School of Medicine, Nagoya, Japan

**Keywords:** rat, channelrhodopsin-2, pain, excitatory postsynaptic current, feed-forward inhibition

## Abstract

A large majority of neurons in the superficial layer of the dorsal horn projects to the lateral parabrachial nucleus (LPB). LPB neurons then project to the capsular part of the central amygdala (CeA; CeC), a key structure underlying the nociception-emotion link. LPB-CeC synaptic transmission is enhanced in various pain models by using electrical stimulation of putative fibers of LPB origin in brain slices. However, this approach has limitations for examining direct monosynaptic connections devoid of directly stimulating fibers from other structures and local GABAergic neurons. To overcome these limitations, we infected the LPB of rats with an adeno-associated virus vector expressing channelrhodopsin-2 and prepared coronal and horizontal brain slices containing the amygdala. We found that blue light stimulation resulted in monosynaptic excitatory postsynaptic currents (EPSCs), with very small latency fluctuations, followed by a large polysynaptic inhibitory postsynaptic current in CeC neurons, regardless of the firing pattern type. Intraplantar formalin injection at 24 h before slice preparation significantly increased EPSC amplitude in late firing-type CeC neurons. These results indicate that direct monosynaptic glutamatergic inputs from the LPB not only excite CeC neurons but also regulate CeA network signaling through robust feed-forward inhibition, which is under plastic modulation in response to persistent inflammatory pain.

nociception signals converging on the spinal dorsal horn or spinal trigeminal nucleus from the periphery are first integrated in these structures and then conveyed to the brain loci underlying the central processing of nociceptive information, thereby triggering acute nociception-associated responses and chronic changes associated with pain, such as chronic pain-related emotional complications (Hashmi et al. 2013). Of these structures, the lateral parabrachial nucleus (LPB), located in the pons, has been identified as one of the primary targets, in addition to the thalamic nuclei, of nociception-specific ascending neurons in the superficial layer of the dorsal horn and spinal trigeminal nucleus in rodents. For example, ∼95% of ascending neurons in lamina I of the rat lumbar horn target the LPB (Todd 2010). Importantly, a large portion of these ascending fibers projecting to various structures has axon collaterals projecting to the LPB, suggesting that a “carbon copy” of most of the nociceptive information sent from the spinal cord to a specific brain site is also sent to the LPB (Todd 2010).

The importance of this spino (trigemino)-parabrachial projection in terms of pain signaling is strengthened further by accumulating lines of evidence indicating that neurons in the LPB then project to the capsular part of the central amygdala (CeA; CeC) and convey the nociceptive signals (Dong et al. 2010; Gauriau and Bernard 2002; Hunt and Mantyh 2001; Jasmin et al. 1997; Sarhan et al. 2005; Todd 2010). In addition to tracing studies demonstrating a direct connection from the LPB to the CeC and the lateral part of the CeA (CeL) (Basbaum et al. 2009; Bernard et al. 1993; Sarhan et al. 2005), evidence indicates the following: *1*) that 78% of CeC neurons are excited by noxious stimulation in anesthetized rats (Bernard et al. 1992; Neugebauer et al. 2004); *2*) that phosphorylated ERK is increased in CeC neurons in response to formalin-induced inflammatory pain (Carrasquillo and Gereau 2007); and *3*) that CeC neurons show robust synaptic potentiation in various pain models (Adedoyin et al. 2010; Cheng et al. 2011; Han and Neugebauer 2004; Ikeda et al. 2007; Nakao et al. 2012; Neugebauer et al. 2004; Ochiai et al. 2012). On the basis of these lines of evidence, the CeC is named the “nociceptive amygdala” and is thought to play important roles in receiving information for harmful events, such as inflammation and tissue damage. Such a function would be essential in the role of the CeA in associating (potentially) harmful events and emotional memory (Ehrlich et al. 2009; Gozzi et al. 2010; Han et al. 2015; Li et al. 2013; Sato et al. 2015; Watabe et al. 2013; Wilensky et al. 2006). Indeed, recent studies indicate that optogenetic activation of the intra-CeA terminals of fibers arising from the LPB (Sato et al. 2015) and activation of the calcitonin gene-related peptide (CGRP) containing neurons projecting to the CeA (Han et al. 2015) are sufficient to cause associative fear/threat learning.

However, it remains largely undetermined how this nociceptive information arising from the LPB subsequently affects the network activity of the CeA. Do only specific types of CeC neurons receive direct LPB inputs? Do CeC neurons, which are mostly inhibitory, receiving excitatory inputs from the LPB, then affect (inhibit) other neurons in the CeC and other subnuclei in the CeA? Do CeC neurons really receive monosynaptic, direct excitatory inputs from the LPB and not through an intermediary of relaying excitatory neurons or other neighboring afferent pathways? Finally, is it really these synapses between fibers of LPB origin and CeC neurons that show synaptic potentiation in persistent pain models? These issues have been almost impossible to address directly, primarily because of limitations in the experimental methods for selective stimulation of the fibers arising from the LPB in slice preparations. As such, in the analysis of synaptic transmissions at LPB-CeC synapses, using electrical stimulation in pain and fear models (Neugebauer et al. 2003; Watabe et al. 2013), there were three kinds of unavoidable risks: *1*) stimulating afferent fibers from non-LPB structures (Sah et al. 2003) passing near the electrode; *2*) stimulating only a subset of fibers remaining in the slice and not stimulating the fibers taking distinct entrance routes to the CeC (Sarhan et al. 2005); and *3*) directly stimulating local GABAergic neurons, which would be indistinguishable from the polysynaptically activated inhibitory inputs from the GABAergic neurons in the CeC (Ehrlich et al. 2009).

Recent advances in optogenetic techniques have enabled the avoidance of some of these risks. In addition to the selective activation of a subpopulation of neurons with specific promotor activity (“gene expression-specific” activation), localized microinjection of optogenetic vectors has enabled the selective activation of specific axons (“projection-specific” activation). In particular, optogenetics has also been applied successfully to the analysis of the synaptic properties of the connections of long-distance projections in slice preparations [for review, see Betley and Sternson (2011)]. Such applications are particularly powerful for recording synaptic transmissions in cases where the origin and target nuclei are distant, and the specific afferent fibers near the target cannot be identified visually for the placement of stimulation electrodes. Recently, two reports demonstrated an involvement of the LPB-CeC pathway in fear/threat acquisition, independently using gene expression- and projection-specific optogenetic activation of this pathway (Han et al. 2015; Sato et al. 2015).

Here, we report, using a projection-specific optogenetic approach, that the inputs with an origin in the LPB and surrounding structures: *1*) monosynaptically excite most types of CeC neurons, both in coronal and horizontal slices; *2*) trigger, in addition to the monosynaptic excitatory responses, large and long-lasting, feed-forward inhibition; and *3*) are potentiated in a persistent inflammatory pain model in a manner dependent on the electroresponsive properties of postsynaptic CeC neurons.

## MATERIALS AND METHODS

### 

#### Injection of an adeno-associated virus vector carrying the ChR2 gene into the LPB.

The manipulation of the animals was approved by the Institutional Animal Care and Use Committee of The Jikei University and conformed to the Guidelines for the Proper Conduct of Animal Experiments of the Science Council of Japan (2006). Four- to five-week-old male Wistar rats were anesthetized initially with sodium pentobarbital (50 mg/kg body wt ip) and placed in a stereotaxic frame. Anesthesia was then maintained with isoflurane (1.5–2% in 100% O_2_). An incision was made to expose the skull surface. A small hole was drilled in the skull, and the underlying dura was removed. With the use of a 2- or 10-μl Hamilton syringe with a 30-gauge needle, 0.75–1.0 μl virus suspension, containing an adeno-associated virus (AAV), encoding modified channelrhodopsin-2 [ChR2(H134R)], fused to enhanced yellow fluorescent protein (EYFP) under the control of the human synapsin promotor [AAV5-hSyn-ChR2(H134R)-EYFP; Penn Vector Core, Philadelphia, PA], was delivered at a rate of 75 nl/min into the LPB. The following coordinates from the bregma were used for the LPB: 15° anteroposterior angle, 6.6-6.5 mm posterior to the bregma, 2.2–2.3 mm lateral to the midline, and 7.4-7.1 mm dorso-ventral. In a subset of rats (*n* = 6), 0.83% fluorescent microspheres (FluoSpheres; 0.04 μm, 565/580; Thermo Fisher Scientific Life Sciences, Waltham, MA) were added to the AAV solution injected into the LPB bilaterally (5 rats) or unilaterally (1 rat) to analyze the relationship between LPB virus injection and EYFP expression in the LPB and CeC (11 sides from 6 rats; see *Verification of injection sites in the LPB* below). After completion of the injection procedure, the skin was sutured with 4-0 silk threads, and the rats were replaced in their home cages.

#### Verification of injection sites in the LPB.

In this study, we used projection-specific optogenetic activation of the LPB-CeC pathway rather than expression-specific activation. For this reason, we carefully examined the regions of virus injection as below. First, we confirmed the expression pattern of EYFP in the LPB at 2 wk postinjection. Under deep isoflurane anesthesia, the brain stem was removed and dipped in a fixative solution [4% paraformaldehyde (PFA) in phosphate buffer (PB; 0.1 M, pH 7.5)] and kept for >1 day. A series of 100 μm-thick coronal slices containing the LPB was made to visualize the somatic and membrane expression of ChR2-tagged EYFP. The slices were stained with propidium iodide (PI), and EYFP and PI fluorescence was observed using a confocal microscope (FV-300; Olympus, Tokyo, Japan). Second, to examine the relationship between the injection site and EYFP expression, solutions containing both the AAV vector and fluorescent microspheres were injected into the bilateral (5 rats) and unilateral (1 rat) CeC (a total of 11 sides). At 7–9 wk postinjection, coronal slices containing the LPB (100 μm thick) and those containing the amygdala (300 μm thick) were made from these rats, and the fluorescence of EYFP and microspheres in the LPB (BX-63; Olympus) and EYFP fluorescence in the CeA (BX-51WI; Olympus) were visualized. To minimize ChR2 activation (activation peak at 470 nm) during fluorescence observation, an excitation filter with a steep and narrow bandpass at 490–500 nm (UMYFPHQ; Olympus) and a 25% neutral density filter (U-25ND25; Olympus) were used. In addition, the duration of illumination was kept to a minimum with an image-accumulating device (InvestiGater; DAGE-MTI, Michigan City, IN). When microsphere-labeled injection sites were identified within or in the vicinity of the LPB, EYFP expression was found in the regions from the LPB, partly the Kölliker-Fuse (KF) and more ventral region of the pons. In the rostral part of the brains from the same rats, heavy EYFP expression was observed in the ipsilateral CeC from the same side of the same rat (10 out of 11 cases of LPB injection). Such a pattern is quite similar to the pattern of enhanced green fluorescent protein expression in mice after an intra-LPB injection of the EGFP-expressing vector (Allen Brain Atlas: experiment 268415561). In contrast, when microsphere labeling was found medially to the LPB or even to the superior cerebellar peduncle (scp), EYFP expression was also found medially to the LPB and scp. In such cases, EYFP fluorescence was much weaker in the CeC (such a case was found in 1 out of 11 injections and analysis). Third, the expression of EYFP was postconfirmed in the brain stem of the rats, from which the electrophysiological recordings from the amygdala were made. After removing the forebrain for making slices containing the amygdala, the brain stem block was removed and dipped in 4% PFA solution for >1 day. Later, 100 μm coronal sections were made and visualized to confirm successful injection in the LPB. We discarded the electrophysiological data from rats in which extensive EYFP expression was observed in the regions medial to the LPB, according to the results of preassessments. This judgment was made independently by two different examiners.

#### Slice preparation for electrophysiological recordings.

At 5–7 wk after virus injection, rats were prepared for electrophysiological recordings, according to procedures previously described from our laboratory (Ikeda et al. 2007; Nakao et al. 2012) and those adapted for aged adult brains (Zhao et al. 2011). The rats were first perfused transcardially with ice-cold cutting solution under isoflurane anesthesia (5% in 100% O_2_) and killed. The brain was then removed, and a block of the forebrain containing the amygdala was dissected out and cut at the midline in ice-cold cutting solution composed of (in mM) the following: 2.5 KCl, 0.5 CaCl_2_, 10 MgSO_4_, 1.25 NaH_2_PO_4_, 2 thiourea, 3 sodium pyruvate, 93 *N*-methyl-d-glucamine, 20 HEPES, 12 *N*-acetyl-l-cysteine, 25 d-glucose, 5 l-ascorbic acid, and 30 NaHCO_3_, equilibrated with 95% O_2_ + 5% CO_2_ (osmolality, ∼290 mosmol/kgH_2_O). The pH of the solution was titrated to 7.1–7.5 with concentrated HCl. A block of the mesencephalon containing the LPB was also dissected out and fixed in 4% PFA for later histological observation (see above). The dissected hemisphere containing the amygdala was secured on the cutting stage of a vibrating blade slicer (DTK-1000 or PRO 7; Dosaka EM, Kyoto, Japan) with the rostral end upward to cut coronal slices. To cut horizontal slices, the dissected hemisphere was secured on the cutting stage with the ventral end upward. The dissected hemisphere was embedded immediately in a 37°C agarose solution (1.6%; Sigma, St. Louis, MO), which was solidified by immediate cooling by covering it with ice-cold cutting solution, and brain slices of 300 μm thickness were prepared. The slices were first incubated in a holding chamber with a constant flow of cutting solution at 34°C for 15 min. After this initial recovery period, the slices were transferred to another holding chamber containing artificial cerebrospinal fluid (ACSF) composed of (in mM) the following: 119 NaCl, 2.5 KCl, 2 CaCl_2_, 2 MgSO_4_, 1.25 NaH_2_PO_4_, 12.5 D-glucose, 5 l-ascorbic acid, 2 thiourea, 3 sodium pyruvate, and 26 NaHCO_3_ (pH ∼7.3, bubbled with 95% O_2_ + 5% CO_2_; osmolality, ∼300–310 mosmol/kgH_2_O) at room temperature (20–25°C) until electrophysiological recording. Each slice was transferred to a recording chamber (∼0.4 ml vol) and fixed with nylon grids to a platinum frame. The slice was submerged in and superfused continuously at a rate of 1–2 ml/min with the ACSF described above. Except for the experiments analyzing inhibitory responses in coronal and horizontal slices (see Figs. 6 and 7, respectively), the ACSF also contained 100 μM picrotoxin.

#### Patch-clamp recordings in the CeC, CeM, and LPB.

Neurons in the CeC and medial subdivision of the CeA (CeM) were identified visually under an upright microscope (BX-51WI; Olympus) with oblique illumination. Images from living slices during the course of electrophysiological recordings were captured using a charge-coupled device camera (IR-1000; DAGE-MTI) and stored digitally on a computer. Whole-cell transmembrane currents were recorded from neurons in the LPB (see Fig. 2) and CeA (see Figs. 3–8). Patch-clamp electrodes were made from borosilicate glass pipettes (1B120F-4; World Precision Instruments, Sarasota, FL). The tip resistance of the electrode was 3–8 MΩ. The composition of the internal solution was (in mM) the following: 120 potassium gluconate, 6 NaCl, 1 CaCl_2_, 2 MgCl_2_, 2 ATP Mg, 0.5 GTP Na, 12 phosphocreatine Na_2_, 5 EGTA, and 10 HEPES hemisodium (pH 7.3, as adjusted with KOH; osmolality, ∼290 mosmol/kgH_2_O). In the experiments analyzing inhibitory responses, the internal solution contained (in mM) the following: 120 cesium gluconate, 6 NaCl, 10 HEPES hemisodium, 12 phosphocreatine Na_2_, 5 EGTA, 1 CaCl_2_, 2 MgCl_2_, 2 ATP Mg, and 0.5 GTP Na (pH 7.4, as adjusted with CsOH; osmolality, ∼290 mosmol/kgH_2_O). Light-evoked excitatory postsynaptic currents (leEPSCs) were recorded at a holding potential of −60 mV. Input resistance, resting membrane potential, and whole-cell capacitance were measured immediately after the establishment of whole-cell mode by membrane rupture. Whole-cell currents were recorded using an Axopatch 700B amplifier (Molecular Devices, Sunnyvale, CA), filtered at 2 kHz, and digitized at 4 or 10 kHz (membrane potentials) or 10 kHz (membrane currents) with 16-bit resolution using a PowerLab interface (ADInstruments, Colorado Springs, CO). Oblique illumination images and epifluorescence images were captured using the same camera without moving stage and the microscope position, and digitally captured images were overlaid in Photoshop software (version 5.5; Adobe Systems, San Jose, CA), with modification only of the brightness and contrast. All recordings were made at room temperature (20–25°C).

Electrophysiological recordings of the light-evoked responses in LPB neurons were made at 2 wk after virus injection in coronal slices prepared and visualized in an almost-similar manner to that described above for preparing CeA slices. Each slice was transferred to a recording chamber and fixed with nylon grids to a platinum frame. The slice was submerged in and superfused continuously at a rate of 1–2 ml/min with ACSF containing kynurenic acid (Kyn; 3 mM) to block glutamatergic transmission. Whole-cell voltage-clamp recordings and current-clamp recordings were obtained from visually identified EYFP-expressing neurons in the LPB. Light-activated currents were recorded at a holding potential of −60 mV, and light-evoked potentials were recorded at the resting potential.

#### Light stimulation of the slices.

ChR2 was activated using a high-power light-emitting diode (LED) illumination system (465 nm, LEX-2B; Brainvision, Tokyo, Japan) controlled by Master-8 (A.M.P.I., Jerusalem, Israel). Light duration was determined with Master-8. Light intensity at the level of the recording chamber was measured using a digital optical power meter (9742-10/3664; Hioki, Nagano, Japan). Illumination was delivered to the whole field through a 40×, 0.8 numerical aperture objective (LumPlan FL N; Olympus). According to the results, seen in Fig. 3, we used 5 ms duration and 9.6 mW/mm^2^ intensity (full-power illumination with LEX-2B without neutral density filter), unless stated otherwise. The field iris diaphragm of the fluorescent path of BX-51WI was kept fully open for the whole experiment.

#### Classification of CeC neurons according to their electroresponsive properties.

It has been suggested that the firing pattern of CeC neurons is related to the role of specific neurons in the CeA network (Haubensak et al. 2010). The electroresponsive properties of the neurons, such as firing pattern, were classified using action potential responses under current-clamp recordings, according to methods described previously (Amano et al. 2012; Chieng et al. 2006; Haubensak et al. 2010).

For all cells in which electroresponsive properties were analyzed, a continuous current was injected manually to keep the resting membrane at approximately −60 mV. Action potentials were triggered by injecting steps of a depolarizing pulse (500 ms; 20 pA step), given immediately after a fixed hyperpolarizing pulse (2 s) that hyperpolarized the neuron below and closest to −80 mV to de-inactivate the inactivating channels sufficiently (e.g., see Fig. 8).

“Low-threshold bursting” (LTB) neurons were characterized by short bursting action potentials immediately after above-threshold depolarization and the rapid accommodation of this firing. This short bursting was not observed when a prehyperpolarizing pulse was not applied, suggesting an involvement of T-type voltage-dependent Ca^2+^ channels. “Late firing” (LF) neurons were characterized by the appearance of an inflection point in the membrane potential trajectory (fast capacitive depolarization, followed abruptly by a slow depolarizing phase) and a long latency for the first action potential generation (AP delay) after depolarization, due to this slow depolarizing phase. AP delay was measured for the action potentials generated at the smallest depolarizing step that would theoretically bring the membrane potential [R_IN_ (GΩ) × 20 (pA) × step number + −60 (mV)] to a level above and closest to −45 mV, where R_IN_ is the break-in membrane resistance measured at −60 mV immediately after membrane rupture, and step number is an integer from 1 to 10 (depending on the membrane potential attained). When this procedure did not induce the cell to fire (*n* = 4 neurons), the latency at the smallest depolarizing step that induced the cell to fire was measured. The LF neurons showed an AP delay >85 ms. The remaining neurons that did not meet the criteria for LTB and LF neurons were classified as “regular spiking” (RS) neurons. These neurons showed regular firing during depolarization (Table 1).

**Table 1.** Basic properties of CeC neurons with 3 firing patterns from saline- or formalin-treated rats

Cell Type	Number of Rats	Number of Neurons (%)	V_rest_, mV (Range)	R_in_, MΩ (Range)	AP Delay, ms (Range)	AP Threshold, mV (Range)
Saline						
LTB	3	4 (10)	−56.9 ± 4.6	305.0 ± 120.6	29.4 ± 2.1	−50.6 ± 3.0
			(−50.0 to −70.4)	(87.6–565.0)	(24.8–33.3)	(−45.4 to −57.2)
RS	6	20 (49)	−59.8 ± 2.9	237.3 ± 24.7	44.2 ± 4.0	−45.7 ± 1.2*
			(−38.1 to −79.3)	(79.1–468.1)	(20.8–76.5)	(−32.7 to −56.5)
LF	6	17 (41)	−63.5 ± 2.5	225.4 ± 25.2	218.7 ± 24.7†	−37.3 ± 1.1†
			(−36.1 to −75.8)	(98.7–442.8)	(88.8–395.3)	(−30.6 to −44.2)

Formalin						
LTB	7	11 (19)	−59.4 ± 2.9	300.0 ± 62.8	38.4 ± 5.2	−46.7 ± 1.7‡
			(−41.0 to −70.6)	(81.9–819.2)	(15.3–70.8)	(−35.5 to −55.1)
RS	10	25 (44)	−64.5 ± 2.1	206.9 ± 19.7	52.1 ± 4.5	−41.4 ± 1.5
			(−39.4 to −75.5)	(93.1–512.0)	(21.3–120.0)	(−20.1 to −53.2)
LF	10	21 (37)	−62.1 ± 3.3	220.1 ± 32.3	231.1 ± 27.5§	−37.3 ± 1.3
			(−35.9 to −79.0)	(48.9–744.7)	(93.3–440.0)	(−21.5 to −47.3)

Data are expressed as the means ± SE. There was a significant difference between the AP threshold of RS neurons in the saline group and in the formalin group.

* *P* < 0.05, Mann-Whitney *U*-test. There were significant differences between the AP delay and threshold of LF neurons and those of LTB and RS neurons in the saline group.

† *P* < 0.001, Gabriel's test; AP delay, *F*(2,38) = 34.60; AP threshold, *F*(2,38) = 18.38. In the formalin group, there was a significant difference between the AP threshold of LTB neurons and LF neurons.

‡ *P* < 0.002, Gabriel's test; *F*(2,54) = 7.148. The AP delay of LF neurons was significantly longer than that of LTB and RS neurons in the formalin group.

§ *P* < 0.001, Gabriel's test; *F*(2,54) = 36.36. There was no significant difference in resting potential (V_rest_) and input resistance (R_in_) between the saline and formalin groups and between the different types of neurons.

#### Formalin-induced inflammatory pain model.

The rats were anesthetized with isoflurane (5% in 100% O_2_) briefly during the following intraplantar formalin injections. Inflammation was induced by injecting 50 μl of 5% formalin (37% formaldehyde solution diluted in saline solution, 30-gauge needle) into the intraplantar surface of the left hindpaw. Control rats were injected with 50 μl saline solution (0.9% NaCl) into the left hindpaw. The rats were video monitored for 1 h after injection, and licking time was evaluated for 50 min. At 24 h after injection, the rats were prepared for electrophysiological recordings.

#### Immunohistochemistry.

Immunohistochemical analysis of CGRP expression was performed, according to standard procedures (D'Hanis et al. 2007; Kinoshita et al. 2013). Briefly, at 2 wk after intra-LPB injection of 0.75 μl virus suspension containing 0.33% fluorescent microspheres (FluoSpheres; 0.02 μm, 365/415; Thermo Fisher Scientific Life Sciences), the rats were anesthetized with sodium pentobarbital (50 mg/kg ip) and perfused intracardially with a fixative solution containing 4% PFA, 15% saturated picric acid, and 0.1% glutaraldehyde in 0.1 M PB (pH 7.4). The brain was removed and incubated overnight in the same fixative solution devoid of glutaraldehyde at 4°C. The brain block was incubated for two nights in 20% buffered sucrose at 4°C and cut into 25 μm-thick sections using a cryostat (CM1850; Leica Biosystems, Tokyo, Japan). Every fourth section was used for immunohistochemistry. After washing three times in 0.1 M PB (10 min), the slices were preincubated in 0.1 M PB containing 5% Triton X-100 and 5% normal goat serum (NGS; Sigma) and then incubated for the next 48–72 h in 0.1 M PB containing the primary rabbit antibody against CGRP (1:1,500, C8198; Sigma), 1% NGS, and 5% Triton X-100 at 4°C. After rinsing three times in 0.1 M PB (10 min), the slices were incubated for 90 min with a biotinylated goat anti-rabbit IgG (Vector Laboratories, Burlingame, CA) in 0.1 M PB containing 1% NGS and 5% Triton X-100. After repeated rinsing, they were incubated for 2 h in 0.1 M PB containing 0.1% streptavidin-Cy3 (Thermo Fisher Scientific Life Sciences), 1% NGS, and 5% Triton X-100; rinsed three times in 0.1 M PB (10 min); mounted on glass slides; and embedded in Aqua-Poly (Polysciences, Warrington, PA). Images were captured with an electron-multiplying charge-coupled device-based confocal microscope (iXon Ultra 897, Andor, Belfast, UK; CSU-X1, Yokogawa Electric, Tokyo, Japan; BX-51WI, Olympus) and aligned using Photoshop, version 5.5, with only contrast and brightness adjustments over the whole image. The same procedures were repeated in two rats.

#### Drugs.

TTX and D-(−)-2-amino-5-phosphonopentanoic acid (AP5) were purchased from Tocris (Bristol, UK). 4-Aminopyridine (4AP), Kyn, picrotoxin, and bicuculline (Bic) were purchased from Sigma. Stock solutions of TTX, 4AP, Bic, and AP5 were dissolved in water, kept frozen at −30°C, and then dissolved in ACSF to their final concentration on the day of the experiment.

#### Data and statistical analysis.

The recorded membrane currents and potentials were analyzed offline with IGOR Pro 6 (WaveMetrics, Lake Oswego, OR) using procedures written by F. Kato. Values are expressed as means ± SE. Differences in the values were compared using Student's *t*-test, one-way and two-way ANOVA for repeated measures, Mann-Whitney *U*-test, and Wilcoxon's signed-rank test. For multiple comparisons, probabilities were corrected using Bonferroni compensation. Statistical calculations were made with SPSS 19.0 (IBM, Tokyo, Japan). Differences with *P* < 0.05 were considered significant.

## RESULTS

### 

#### Functional ChR2 expression in LPB neurons.

**Fig. 1.**
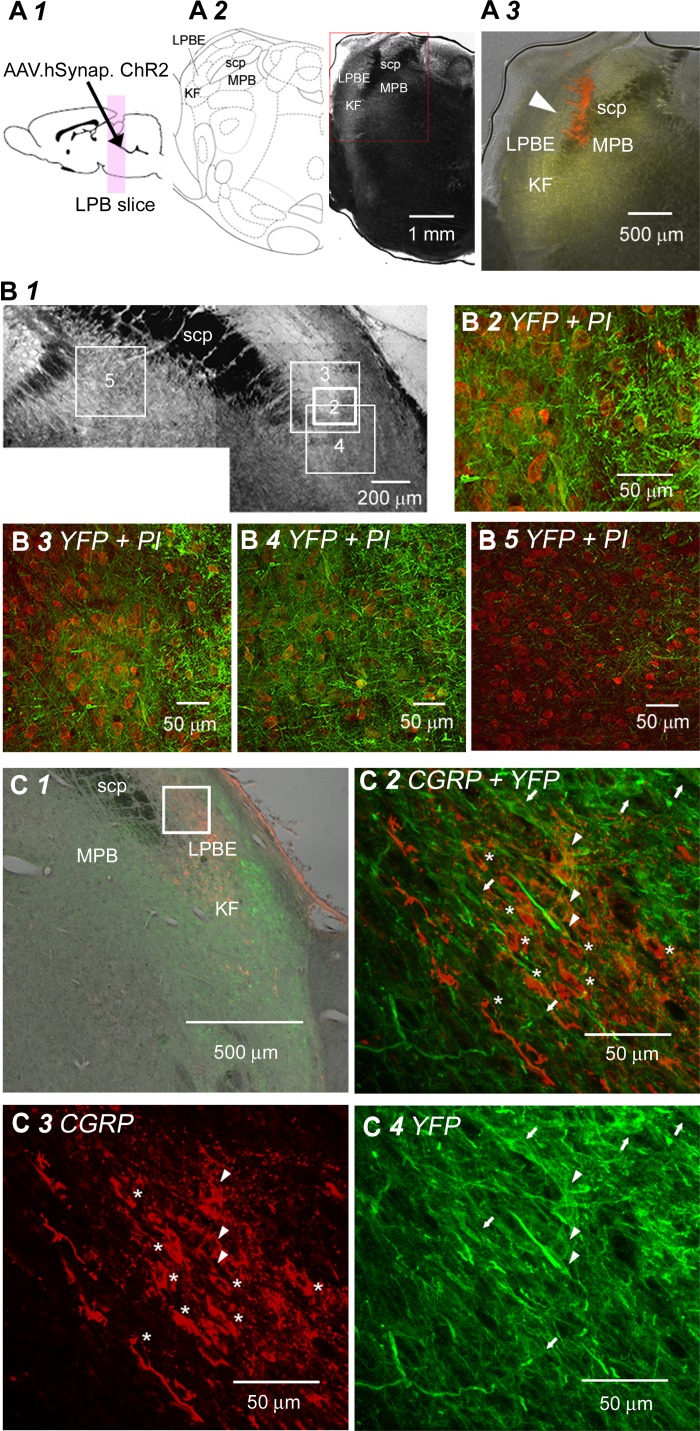
ChR2 expression in LPB neurons. *A*: illustrated rat brain, indicating the experiments presented in *A–C* (*A1*). Illustration of a coronal slice from the rat brain atlas (Paxinos and Watson 2007), showing the LPB and surrounding structures in the coronal plane (*left*) and a representative micrograph of a coronal slice at the same rostrocaudal level containing the LPB (*right*), prepared from a rat with AAV-ChR2(H134R)-EYFP injection into the LPB (*A2*). A higher magnification image showing overlaid EYFP fluorescence (yellow) expression around the LPB (*A3*). The center of the injection site was confirmed with the fluorescence of the microsphere beads (red). The area within the red rectangle in *A2* is shown at a higher magnification in *A3*. The brains were removed and fixed at 62 days (∼9 wk) after virus injection. hSynap., human synapsin promotor; MPB, medial parabrachial nucleus. *B*: representative images of a coronal slice containing the LPB prepared from a rat at 2 wk after intra-LPB injection of AAV-ChR2(H134R)-EYFP (*B1*). Higher magnification images of the LPB showing colocalization of EYFP (green) with PI (red; *B2–B4*). There was no colocalization of EYFP with PI medially to the LPB (*B5*). *C*: image of a coronal slice showing the localization of CGRP-, EYFP-, and double-positive neurons in the LPB and surrounding area (*C1*). The brain was removed and fixed at 2 wk after virus injection. The area within the rectangle in C1 is shown at a higher magnification in *C2–C4*. CGRP-, EYFP-, and double-positive neurons were observed in the LPB. Stars, CGRP-positive neurons; arrows, EYFP-positive neurons; arrowheads, double-positive neurons. [The schemas for experimental setup (Figs. 1*A1*, 1*A2*, 2*A1*, 3*A1*, and 7*A1*) and plots for neuron location (Figs. 7*A4* and 8*G*) are based on the atlas by Paxinos and Watson (2007), used with permission. This was published in *The Rat Brain in Stereotaxic Coordinates* Paxinos and Watson, Copyright Elsevier (2007).]

First, we confirmed that the LPB neurons at the site of viral vector injection expressed EYFP signals. Injection of AAV5-hSyn-ChR2(H134R)-EYFP resulted in EYFP fluorescence at the level of the injection tip, which was confirmed with the fluorescence of the microsphere beads (Fig. 1, *A2* and *A3*). The images in Fig. 1, *B1–B5*, were taken at 2 wk after AAV vector injection. Neurons with EYFP signals, surrounding the soma and also within the soma, were densely found in the external LPB (LPBE; Fig. 1, *B2* and *B3*) and in ventral regions within the LPB slightly dorsal to the KF (Fig. 1*B4*) and not in the regions medial to the scp (Fig. 1*B5*). No EYFP signal was observed in the locus coeruleus in slices with successful injections. Immunostaining for CGRP, a molecule expressed in a large number of CeA-projecting neurons in the LPB (D'Hanis et al. 2007; Dong et al. 2010) in the pontine slices of rats with successful vector injection, indicated that CGRP expression, which was limited to the LPBE and KF in regions dorso-lateral and ventro-lateral to the scp, overlapped with EYFP expression in the LPBE and adjacent regions (Fig. 1*C*). Magnified observations of the LPBE indicated that some of the neurons expressing CGRP also expressed EYFP and vice versa (Fig. 1, *C2*–*C4*). This observation is reminiscent of the relationship between retrograde staining from the CeA and expression of CGRP in LPB neurons of rats (D'Hanis et al. 2007), suggesting that some of the CGRP-positive neurons project to the CeA and vice versa. Very similar and consistent results were obtained in six slices with marked CGRP immunoresponses from a total of 26 slices spanning over the LPB of two rats with bilateral virus injections.

**Fig. 2.**
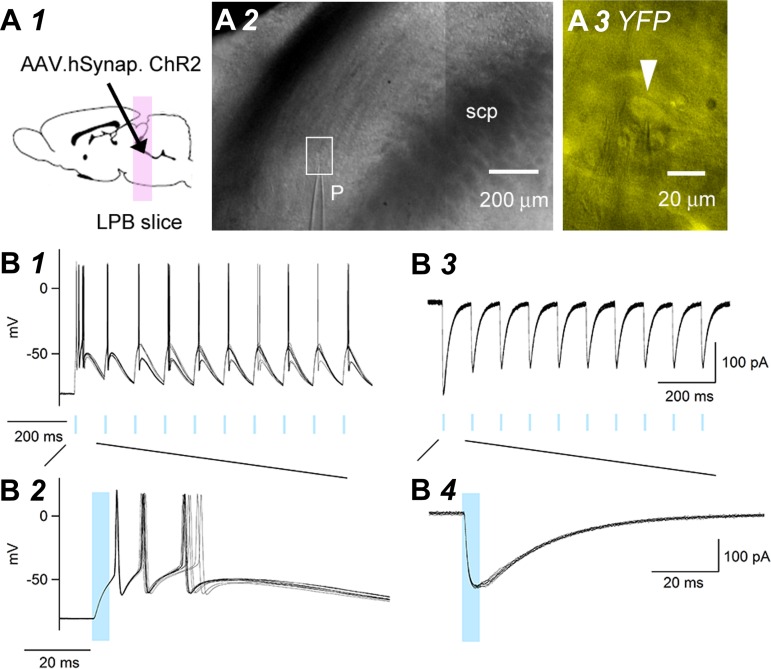
Light-evoked responses in ChR2-positive LPB neurons. *A*: illustrated rat brain, indicating the experiments presented in *A1*. At 2 wk after intra-LPB injection, acute brain slices containing the LPB were prepared for electrophysiological recordings. An image of a coronal slice, indicating the location of the patch recording pipette (p; *A2*). A higher magnification image showing an EYFP-positive LPB neuron (white arrowhead; *A3*). The rectangle in *A2* shows the area in *A3* at a higher magnification. *B*: representative traces of the responses evoked by blue light stimulation (duration 5 ms, 10 Hz), recorded from an EYFP-positive LPB neuron. *B1*: effects of light stimulation on the membrane potential at resting potential; consecutive responses (overlaid). *B2*: the first action potential at a faster sweep. *B3*: effects on the membrane current recorded at a holding potential of −60 mV. Overlaid consecutive responses. *B4*: the first inward current at a faster sweep. The blue rectangles show the period of light on. All recordings were made in the presence of Kyn (3 mM). [The schemas for experimental setup (Figs. 1*A1*, 1*A2*, 2*A1*, 3*A1*, and 7*A1*) and plots for neuron location (Figs. 7*A4* and 8*G*) are based on the atlas by Paxinos and Watson (2007), used with permission. This was published in *The Rat Brain in Stereotaxic Coordinates*, Paxinos and Watson, Copyright Elsevier (2007).]

Whole-cell patch-clamp recordings from six LPB neurons were made from these EYFP-positive (Fig. 2*A3*) neurons with healthy morphology under visual guidance at 2 wk after injection (Fig. 2, *A1–A3*). These cells had resting potentials of −76.6 to −58.7 mV and did not show spontaneous firing at rest. Blue light stimulation of these neurons immediately depolarized the cells (Fig. 2*B1*), which induced the cells to generate action potentials (Fig. 2*B1*). Latency from the onset of the light to that of depolarization was within 500 μs, with very small variation over trials (Fig. 2*B2*). Similarly, a highly stable inward current was triggered consistently by blue light stimulation under voltage-clamp recording (holding potential at −60 mV; Fig. 2, *B3* and *B4*; an overlay of 10 consecutive trials). These data suggest that LPB neurons expressing EYFP expressed functional ChR2 that was activated by blue light stimulation at ∼2 wk after AAV vector transfection.

#### CeC neurons show robust leEPSCs.

**Fig. 3.**
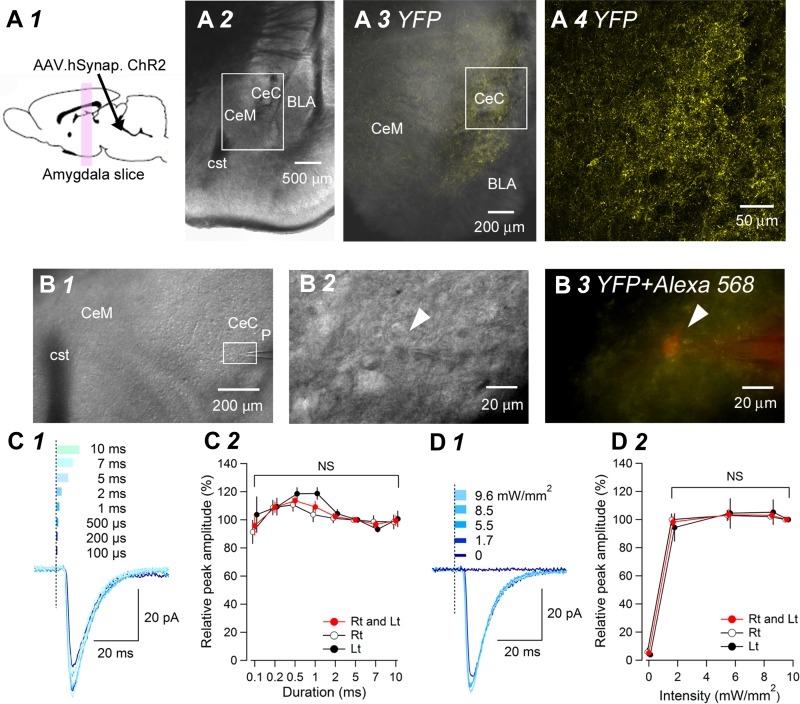
Effects of light duration and intensity on light-evoked EPSCs in CeC neurons in coronal slices. *A*: schema illustrating the experimental setup for the results shown in *A1*. At 6 wk after intra-LPB injection of AAV-ChR2(H134R)-EYFP, acute brain slices, containing the amygdala, were prepared for electrophysiological recordings. A representative differential interference contrast image of a coronal slice containing the amygdala (*A2*) and a higher magnification image showing the fibers expressing EYFP (yellow) in the CeC (*A3*). Note the high-density, EYFP-positive fibers in the CeC (*A3* and *A4*). The rectangles in *A2* and *A3* correspond to the areas in *A3* and *A4*, respectively, at a higher magnification. cst, commissural stria terminalis; BLA, basolateral amygdaloid nucleus. *B*: image of a coronal slice used for patch-clamp recording. *B1*: the position of the recording pipette (p) in the CeC. *B2*: the recorded CeC neuron (white arrowhead) and recording pipette at a higher magnification. *B3*: an overlaid image of Alexa 568 (red) injected into the recorded CeC neuron (white arrowhead) via pipette solution and EYFP (yellow). The rectangle in *B1* shows the area in *B2* and *B3* at a higher magnification. *C*: effects of changing light duration on leEPSC amplitude. *C1*: averaged leEPSC waveforms (*n* = 10 responses) recorded in a representative CeC neuron. Each trace in a distinct color is the response to light stimulation with a distinct duration (durations are shown as the width of the rectangles with distinct colors). *C2*: the relationship between light duration (abscissa) and normalized leEPSC amplitude (ordinate). The leEPSC amplitude evoked by 5 ms light stimulation was used as the 100% value for each neuron (means ± SE of 11 neurons). Light intensity was consistently 9.6 mW/mm^2^. There was no significant difference in leEPSC amplitude between the neurons from the right (Rt) and left (Lt) CeC. Thus we combined the results from the right and left sides for analysis. NS, not significantly different (*P* = 0.076, 1-way ANOVA with repeated measures, F[1.79,17.91] = 3.08). Red, relative peak amplitude recorded from both sides; white, from the right side; black, from the left side. *D*: effects of changing light intensity on leEPSC amplitude. *D1*: averaged leEPSC waveforms (*n* = 10 responses) recorded in a representative CeC neuron. Each trace in blue with a distinct tint and shade shows the response to light stimulation with a distinct intensity (intensities are shown as the height of the rectangles in blue with distinct tints and shades). *D2*: the relationship between light intensity (abscissa) and normalized leEPSC amplitude (ordinate). The leEPSC amplitude evoked by 9.6 mW/mm^2^ intensity light stimulation was used as the 100% value for each neuron (means ± SE of 10 neurons). Light duration was consistently 5 ms. There was no significant difference in the leEPSC amplitude between neurons from the right side and those from the left side. The total values of leEPSC amplitude recorded from both sides were not significantly dependent on changes in light intensity. NS, not significantly different (*P* = 0.136, 1-way ANOVA with repeated measures, F[3,27] = 2.01). Red, relative peak amplitude recorded from both sides; white, from the right side; black, from the left side. [The schemas for experimental setup (Figs. 1*A1*, 1*A2*, 2*A1*, 3*A1*, and 7*A1*) and plots for neuron location (Figs. 7*A4* and 8*G*) are based on the atlas by Paxinos and Watson (2007), used with permission. This was published in *The Rat Brain in Stereotaxic Coordinates*, Paxinos and Watson, Copyright Elsevier (2007).]

We prepared acute brain slices containing the amygdala subnuclei at 5–7 wk after viral injection into the LPB (Fig. 3*A1*). In all animals with successful AAV vector injections (see materials and methods for this criterion), fibers with EYFP fluorescence were observed in the CeC and CeL at a high density (Fig. 3, *A3* and *A4*). We made whole-cell patch-clamp recordings from CeC neurons found amid the EYFP-positive fibers and recorded membrane current responses to blue light stimulation at a holding potential of −60 mV (Fig. 3, *B1–B3*). Blue light stimulation consistently produced robust leEPSCs (Fig. 3*C*) in most of the neurons recorded from slices with a dense fiber appearance with EYFP in the CeC region.

The following two types of evidence suggest that blue light illumination sufficiently activated almost the full set of axons and terminals expressing ChR2 in each slice. First, we assessed the influence of light duration on leEPSC amplitude. Even stimulation with a duration of 100 μs (i.e., duration of the light-on pulse = 100 μs) could successfully trigger leEPSCs (Fig. 3*C1*). The relationship between light duration and leEPSC amplitude (Fig. 3*C2*) indicated that leEPSC amplitude was not significantly dependent on light duration if the duration were longer than 0.1 ms (Fig. 3*C*; *P* = 0.076 for 0.1–10 ms, F[1.79,17.91] = 3.08, 1-way ANOVA with repeated measures; *n* = 11 neurons from 3 rats for the effect of duration on actual leEPSC amplitude after Greenhouse-Geisser correction). According to this result and taking the safety margin into consideration, we stimulated the slices with a light duration of 5 ms in the following experiments, which yielded stable and optimal responses.

We next assessed the effect of changing light intensity on leEPSC amplitude. Contrary to our expectations, changes in light intensity did not essentially affect the amplitude of leEPSCs in our experimental conditions (Fig. 3*D*; 1.7–9.6 mW/mm^2^, *P* = 0.136, F[3,27] = 2.01, 1-way ANOVA with repeated measurements, effect of intensity; *n* = 10 neurons from 3 rats). As the amplitude of leEPSCs would be proportional to the number of activated fibers making synaptic contact with the neuron being recorded, this lack of sensitivity of leEPSC amplitude to light intensity suggests that the vast majority of afferent fibers synapsing to the CeC neurons was activated to generate action potentials in this experimental condition. During this series of experiments, light stimulation (>1.7 mW/mm^2^ intensity) was delivered repeatedly, 60–99 times (mean, 72.7 times; *n* = 10), over a period of 24–37 min (mean, 28 min; *n* = 10). leEPSC amplitude at the end of this series of experiments was 100.4 ± 4.7% (*P* = 0.335, paired *t*-test; *n* = 10) of that at the beginning of the experiments, suggesting that repeated delivery of light stimulation for a long period itself does not essentially affect leEPSC amplitude. Thus in the following experiments, we stimulated the slices with 9.6 mW/mm^2^ intensity and 5 ms duration. Surprisingly, with these conditions, we observed only very few “failure” responses in which blue light delivery resulted in no detectable postsynaptic response (e.g., see Fig. 5), suggesting that the light-evoked release was very robust with a high probability. As expected, stimulation with 0 mW/mm^2^ light intensity by adjusting the dial setup of the LED controller resulted in no evoked responses, indicating that the leEPSCs were indeed triggered by light itself.

It has been suggested that the CeA shows functional lateralization (Carrasquillo and Gereau 2007; Kolber et al. 2010). Figure 3, *C2* and *D2*, also indicate the peak amplitude of the responses of the neurons in the right and left CeC. We failed to find any significant difference in leEPSC amplitude between neurons in the right and left CeC at any duration examined (*P* = 0.242 for the effect of the side, 2-way ANOVA for duration and the side; no significant interaction between the duration and the side; *P* = 0.485, after Greenhouse-Geisser correction, 7 and 4 neurons from the right and left CeC neurons, respectively; Fig. 3*C2*) and at any intensity above 1.7 mW/mm^2 ^examined (*P* = 0.393 for the effect of the side, 2-way ANOVA for intensity and the side; no significant interaction between the intensity and the side; *P* = 1.000, 7 and 3 neurons from the right and left CeC neurons, respectively; Fig. 3*D2*). This absence of significant right-left difference in LPB-CeC transmission at the naïve state is in accordance with that in CeA neuron responses to somatosensory stimulation in naïve rats (Goncalves and Dickenson 2012; Ji and Neugebauer 2009). On the basis of this observation, the data from each side were pooled for statistical analysis in the results described hereafter, unless stated otherwise.

#### Illumination location affects leEPSC responses.

**Fig. 4.**
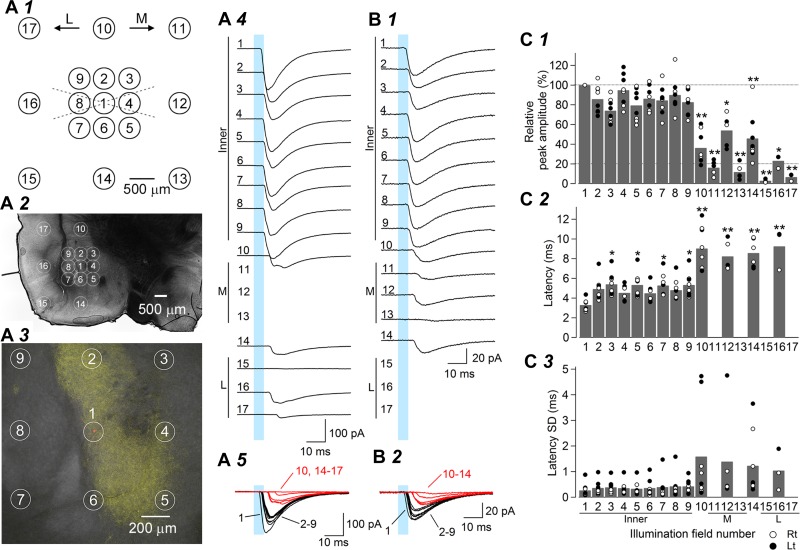
Effects of shifting the illumination field on light-evoked EPSCs in CeC neurons in coronal slices. *A*: schema indicating the locations of LED illumination in coronal slices containing the amygdala (*A1*). Numbered circles indicate the center of the illumination fields. M, medial; L, lateral. Broken lines indicate the positions of the recording pipette (lateral or medial approach, according to slice placement). Illumination field 1 was positioned at the soma of the recorded neuron in the center. Fields 2–9 were at the corners and sides of a square surrounding the recorded neuron (the inner square), whereas fields 11–13 (medial), 15–17 (lateral), 10 (dorsal), and 14 (ventral) were located on the corners and sides of the larger outer square regardless of slice positioning. *A2*: differential contrast interference image of a representative coronal slice containing the amygdala prepared from a rat at 7 wk after intra-LPB injection of AAV-ChR2(H134R)-EYFP. *A3*: a higher magnification image showing overlaid EYFP fluorescence (yellow) and Alexa 568 (red) injected into the recorded CeC neuron. The circles in *A2* and *A3* show the centers of the illumination fields. In the case of the recordings shown in *A2* and *A3*, illumination of the medial part (fields 11–13) was not performed, to avoid interference between the recording pipette and the objective lens (thus no trace is shown in 11–13 in *A4*). *A4*: averaged waveforms of leEPSCs (*n* = 10 consecutive responses) recorded from the neuron shown in *A3*. *A5*: overlaid average traces of leEPSCs (*n* = 10 consecutive responses) showing the differences of leEPSC responses between illumination at the inner (1–9, black traces) and outer (10 and 14–17, red) squares. The blue rectangles in *A4* and *A5* show the timing and duration of blue light stimulation (duration, 5 ms). Inner, responses by illumination at the inner square; M, medial side and corners of the outer square; L, lateral side and corners of the outer square. *B1* and *B2*: the averaged waveforms of leEPSCs from another CeC neuron, which was recorded with the patch pipette approached from the lateral side. *C*: effects of shifting illumination field on leEPSC amplitude (1–10 and 14: *n* = 8; 11–13: *n* = 5; 15–17: *n* = 3; *C1*). Values of the leEPSC amplitude were normalized by that obtained with illumination at the center field (field 1 in *A1*). Summary of the latency (*C2*) and its SD (*C3*) of leEPSCs at different illumination fields (1–10 and 14: *n* = 8; 12: *n* = 5; 16: *n* = 3). The latency and its SD of leEPSCs are not shown for fields 11, 13, 15, and 17, because illumination at these fields often yielded undetectable leEPSCs, making measurement of latency impossible. The bars indicate the mean, whereas the open and filled circles indicate the values recorded from the right and left sides, respectively. *P < 0.02, **P < 0.001, Dunnett's test.

**Fig. 5.**
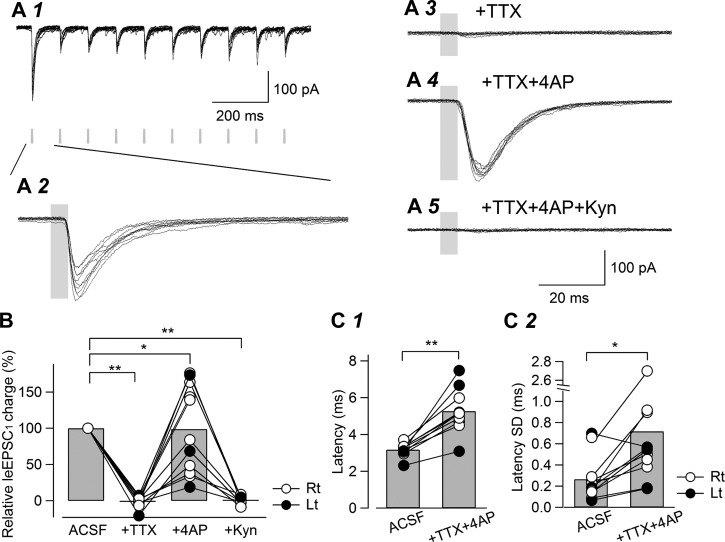
Pharmacological confirmation of monosynaptic LPB-CeC connections in coronal slices. *A*: 10 representative overlaid traces of consecutive responses of a CeC neuron to blue light stimulation (duration, 5 ms, 10 Hz; *A1*). The gray rectangles show the timing and duration of blue light stimulation. *A2*: a time-extended version of the first leEPSC. *A3*: addition of TTX (1 μM) to the ACSF abolished the leEPSCs. *A4*: further addition of 4AP (100 μM) made the leEPSCs reappear. *A5*: further addition of Kyn (3 mM) in the presence of TTX and 4AP completely abolished the leEPSCs. *B*: summary of relative charge movements of the first leEPSC (“Relative leEPSC_1_ charge”) in the absence (ACSF, *n* = 11) or presence (*n* = 11) of TTX, TTX and 4AP (*n* = 11) and in the presence of TTX, 4AP, and Kyn (*n* = 9). Values of the leEPSC charge were normalized by that obtained with ACSF. The bars indicate the mean, whereas the open and filled circles connected with lines indicate the values recorded from the right and left sides, respectively. *P < 0.02, **P < 0.01, Wilcoxon's signed-rank test vs. predrug; *n* = 11. *C*: summary of the difference in latency and its SD of leEPSCs. The leEPSC onset latency in the absence (ACSF, *n* = 11) or presence (*n* = 11) of TTX and 4AP (*C1*). **P < 0.001, paired *t*-test. *C2*: summary of the SD of leEPSC latency (Latency SD) in the absence (ACSF, *n* = 11) or presence (*n* = 11) of TTX and 4AP. *P < 0.05, paired *t*-test. The bars indicate the mean, whereas the open and filled circles connected with lines indicate the values recorded from the right and left sides, respectively.

**Fig. 6.**
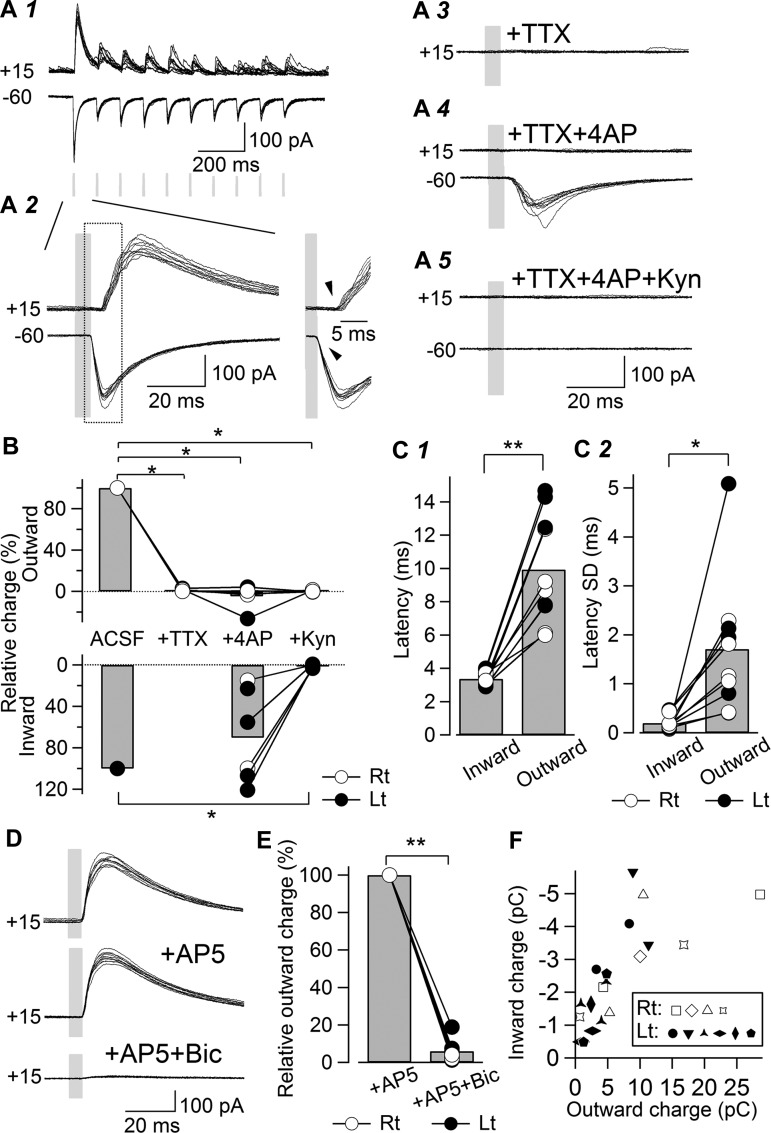
Potent feed-forward inhibition following light-evoked activation of LPB pathways in coronal slices. *A*: representative traces of outward currents recorded at +15 mV (+15) and inward currents at −60 mV (−60) from a representative CeC neuron (*A1*). Overlay of 10 consecutive responses. Blue light stimulation (duration 5 ms, 10 Hz; shown with gray rectangles) evoked a robust inward current at −60 mV (*bottom* traces) and an outward current at +15 mV (*top* traces). *A2*: time-extended version of the responses to the first blue light stimulation. Note that the onset of the outward component was delayed and more varied compared with that of the inward current (arrowheads in the right-most inset). TTX (1 μM) abolished the outward currents (*A3*), and unlike the inward currents, the addition of 4AP (100 μM) did not restore the outward currents (*A4*). *A5*: in the presence of TTX and 4AP, the further addition of Kyn (3 mM) blocked the inward currents. *B*: summary of relative charge movements of the outward and inward currents in response to the first blue light stimulation after each pharmacological manipulation. Total charge movements were normalized by the value obtained with ACSF. The open and filled circles with connections represent the values from the right and left sides, respectively. The outward charge was significantly decreased by the addition of TTX (*n* = 6), TTX and 4AP (*n* = 6), and TTX, 4AP, and Kyn (*n* = 5). The inward charge in the presence of TTX and 4AP was decreased significantly by the further addition of Kyn (*n* = 5). *P < 0.05, Wilcoxon's signed-rank test vs. predrug; *n* = 6. *C1*: summary of the onset latency of outward and inward currents from the beginning of the light pulse. The bars indicate the mean, whereas the line-connected open and filled circles indicate the values from the right and left sides, respectively. Onset latency of the outward currents was significantly longer than that of the inward currents (*n* = 10). **P < 0.001, paired *t*-test. *C2*: summary of the latency SD of outward and inward currents. The bars indicate the mean, whereas the line-connected open and filled circles indicate the values from the right and left neurons, respectively. The latency SD of outward currents was significantly larger than that of the inward currents (*n* = 10). *P < 0.005, paired *t*-test. *D*: representative traces (overlay of 10 responses) of the outward current recorded at +15 mV (+15) from a CeC neuron. *Top* to *bottom*: the outward component recorded with ACSF in the absence of AP5 and Bic, in the presence of AP5 (50 μM), and in the presence of AP5 and Bic (10 μM). The outward current was not essentially affected by the application of AP5, but it was abolished by the further addition of Bic. *E*: summary of the relative charge movements of the first outward current after pharmacological manipulation. The outward charge was normalized by that in the presence of AP5. The open and filled circles represent the values from the right and left neurons, respectively. The relative outward charge was decreased significantly by the addition of Bic (*n* = 9). **P < 0.01, Wilcoxon's signed-rank test. *F*: relationship between the total charge movement of the outward (abscissa) and inward (ordinate) components recorded at +15 mV and −60 mV, respectively, with ACSF. Each marker represents the data from each neuron, and the markers of the same shape represent values from neurons in slices from the same side of the same rat. The open and filled markers represent the values from right and left neurons, respectively. There was a significant correlation between the outward and inward charges (*r* = −0.730, Pearson's correlation coefficient, *P* < 0.001; *n* = 19). This suggests that despite such a variety of leEPSC amplitudes, even from single slices, the total charges of the inward and outward components were correlated linearly.

**Fig. 7.**
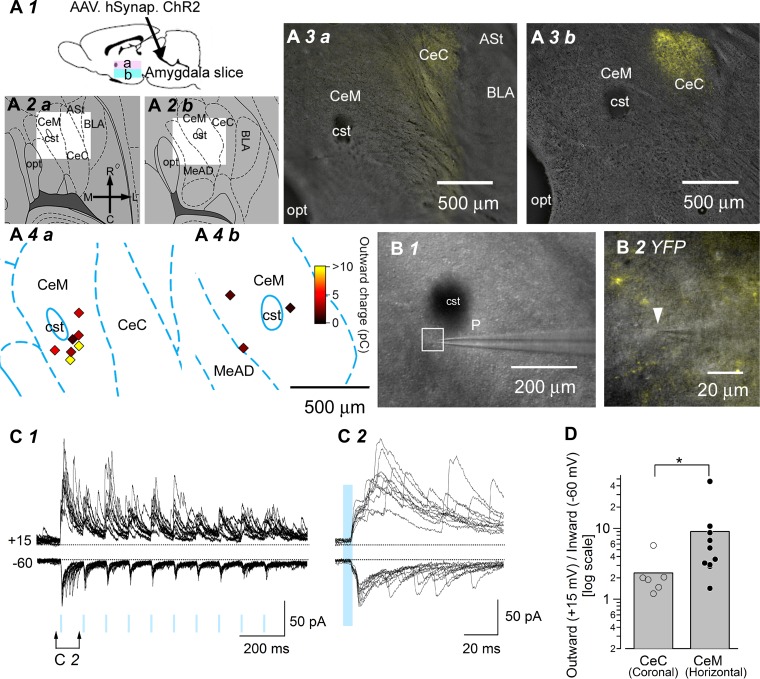
Selective activation of LPB pathways in horizontal slices. *A*: summary of the location of the CeA and recording sites of CeM neurons in horizontal slices. Schema illustrating the experimental setup for the results shown in *A1*. At 6 wk after intra-LPB injection of AAV-ChR2(H134R)-EYFP, horizontal slices containing the amygdala were prepared for electrophysiological recordings. a and b, horizontal slices of dorsal and ventral levels, respectively. *A2*: illustrations of horizontal slices from the rat brain atlas (Paxinos and Watson 2007) showing the CeA and surrounding structures. The unshaded rectangles in the illustrations correspond to the area shown in representative microphotographs of horizontal slices containing the dorsal and ventral portions of the amygdala. *A3*: dense EYFP-positive fibers (yellow) were observed in the CeC. *A4*: illustrations of 2 representative horizontal slices prepared using original drawings from the rat brain atlas. Localization of the recorded CeM neurons (*n* = 10) was plotted as diamonds, and the colors show the values of the outward charges. R, rostral; C, caudal; M, medial; L, lateral; MeAD, medial amygdaloid nucleus, anterior dorsal; opt, optic tract; ASt, amygdalostriatal transition area. *B*: image of a horizontal slice indicating the patch recording pipette (p; *B1*). *B2*: a higher magnification image showing the recorded CeM neuron (white arrowhead). EYFP-positive fibers around the recorded CeM neuron were not as dense as those in the CeC. Note the shadow of the tip of the patch pipette in contact with the neuron being recorded. *C*: representative 10 consecutive, overlaid traces of outward currents recorded at +15 mV (+15) and inward currents at −60 mV (−60) from a representative CeM neuron (*C1*) and the time-extended versions of the responses to the first light stimulation (*C2*). Blue light stimulation (duration 5 ms, 10 Hz; shown with blue rectangles) evoked inward currents at −60 mV and robust outward currents at +15 mV. *D*: summary of the ratio of the outward charge to the inward charge in CeC and CeM neurons. The ratio of the outward charge to the inward charge in CeM neurons (*n* = 10) was significantly larger than that in CeC neurons (*n* = 6). The inward and outward charges of CeC neurons are the same as the values shown in Fig. 6. *P < 0.05, Mann-Whitney *U*-test. [The schemas for experimental setup (Figs. 1*A1*, 1*A2*, 2*A1*, 3*A1*, and 7*A1*) and plots for neuron location (Figs. 7*A4* and 8*G*) are based on the atlas by Paxinos and Watson (2007), used with permission. This was published in *The Rat Brain in Stereotaxic Coordinates*, Paxinos and Watson, Copyright Elsevier (2007).]

**Fig. 8.**
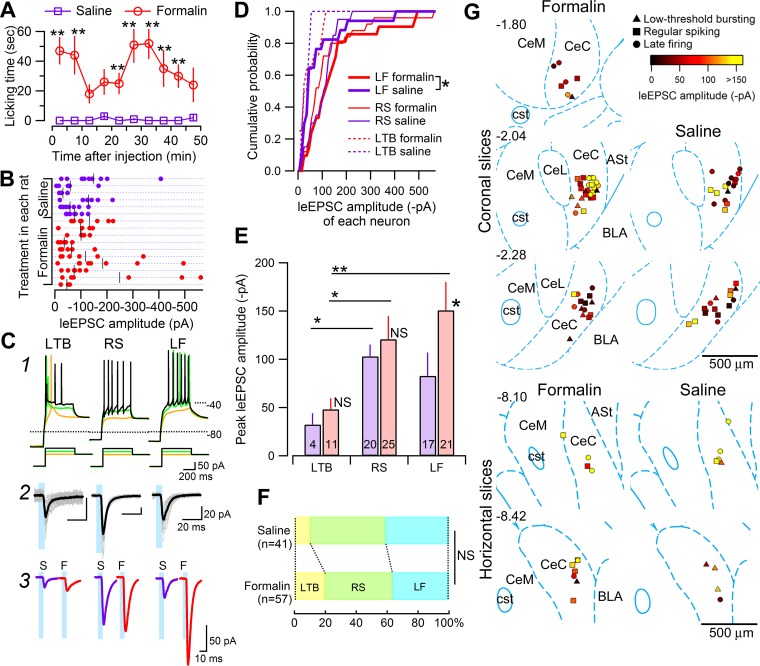
Synaptic potentiation in CeC neurons in the inflammatory pain model revealed by light-evoked activation of LPB fibers. *A*: time course of spontaneous nocifensive behavior (licking) after the injection of saline or 5% formalin solution into the left hindpaw. The licking time of rats injected with the formalin solution (red; 0–10 min: *n* = 9; 10–50 min: *n* = 10) was significantly longer than that of rats injected with saline (violet; *n* = 6) after 0–10 min and 20–45 min. **P < 0.01, Mann-Whitney *U*-test. *B*: summary of the leEPSC amplitude of neurons in the right CeC of rats that received saline (violet; *top*) or formalin (red; *bottom*) at 24 h before slice preparation. One marker represents the result from 1 neuron, whereas the horizontally aligned markers show that they are recorded from slices prepared from single rats. The leEPSC amplitude was calculated on the basis of average leEPSC amplitude over 20 consecutive responses in each neuron. The black vertical bars show the average leEPSC amplitude recorded from each rat (98 cells from 16 rats). *C*: firing patterns of CeC neurons. Neurons were classified into RS, LF, or LTB, according to their firing patterns, in response to depolarizing current injections (duration, 500 ms) delivered after a hyperpolarizing current injection (2 s; *C1*); see text for the detailed criteria. *C2*: clear leEPSC responses to blue light stimulation (duration, 5 ms) were observed in CeC neurons with each firing type. The overlaid traces in gray shown with the blue rectangles indicate 20 consecutive responses to blue light stimulation, whereas the black thick traces show their averages. *C3*: representative averaged waveforms of leEPSCs (*n* = 20 consecutive responses) in CeC neurons with each firing type from saline (S; *left* of each pair)- and formalin (F; *right* of each pair)-treated rats. *D*: cumulative probability of leEPSC amplitude of CeC neurons with each firing type from saline (violet)- and formalin (red)-treated rats. Thick lines, distribution from LF neurons; thin lines, RS neurons; broken lines, LTB neurons. The distribution of leEPSC amplitude for LF neurons from formalin-treated rats (*n* = 21) was significantly different than that from saline-treated rats (*n* = 17; *P < 0.05, Kolmogorov-Smirnov test). *E*: summary of mean and SE (violet and red bars) of leEPSC amplitude recorded from the 3 types of neurons with distinct firing patterns. *P < 0.05, **P < 0.005, Mann-Whitney *U*-test with post hoc Bonferroni correction. NS, not significantly different. The original dataset was the same as that used for the cumulative probability plot in *D*. *F*: the portion of CeC neurons with each firing type recorded from saline- and formalin-treated rats. There was no significant difference between saline (*n* = 41)- and formalin (*n* = 57)-treated rats (*P* = 0.43, χ^2^ test). NS, not significantly different. *G*: summary of the locations of the recorded neurons in formalin- and saline-treated rats (from *left* to *right*, formalin to saline, *n* = 57 and 41, respectively), plotted on 3 representative coronal slices (from *top* to *bottom*, rostral to caudal) and 2 representative horizontal slices (from *top* to *bottom*, dorsal to ventral) (Paxinos and Watson 2007). The marker types show the firing pattern (triangles, LTB; squares, RS; circles, LF), and the color of the markers shows the leEPSC amplitude recorded in each neuron. [The schemas for experimental setup (Figs. 1*A1*, 1*A2*, 2*A1*, 3*A1*, and 7*A1*) and plots for neuron location (Figs. 7*A4* and 8*G*) are based on the atlas by Paxinos and Watson (2007), used with permission. This was published in *The Rat Brain in Stereotaxic Coordinates,* Paxinos and Watson, Copyright Elsevier (2007).]

The responses to light illumination in Fig. 3 (and also see those in Figs. 5–8) were recorded in a configuration, whereby the neuron being recorded was located near the center of the visual field of the microscope. The LED was adjusted so that the center of illumination was located in this center. To examine whether the leEPSC responses depend on the location of illumination on the slice, we moved the location of the objective lens, that is, the location of illumination relative to the neuron soma, while recording postsynaptic responses (Fig. 4). Numbers 1–17 in Fig. 4, *A1–A3*, indicate the location of the centers of illumination relative to the location of the recorded neuron (always number 1). We moved the illumination center by 500 μm in eight directions (numbers 2–9) and by 1,500 μm in eight directions (numbers 10–17; illumination at numbers 11–13 or 15–17 was not performed to avoid interference between the objective lens and the patch pipette, depending on a medial or lateral approach, respectively). In all eight neurons examined, illumination at the inner square (numbers 1–9) gave rise to large and consistently short-latency leEPSCs (Fig. 4, *A4* and *B1* and *A5* and *B2*). However, illumination at the outer square (numbers 10–17) resulted in slow-onset, smaller responses, including failure responses (Fig. 4, *A5* and *B2*). The mean leEPSC amplitude was largest when the slices were illuminated at position number 1 and was significantly smaller than this when the slices were illuminated at the outer square (Fig. 4*C1*). Illumination at the four corner points (numbers 11, 13, 15, and 17) consistently resulted in smaller amplitude leEPSCs than illumination at the side points (numbers 10, 12, 14, and 16). The latency from light-on to leEPSC onset was consistently and substantially longer by 4–5 ms for illumination at the outer (numbers 10, 12, 14, and 16)- than for inner (numbers 1–9)-square illumination (Fig. 4, *A5* and *B2*; also see Fig. 4*C2*; the mean latency for the leEPSCs evoked by illumination at the outer-corner sites, that is, numbers 11, 13, 15, and 17, was not plotted in Fig. 4, *C2* and *C3*, because the leEPSCs were too small or undetectable to measure precisely the latency at these illumination sites). The latency ranged from 3.3 to 5.4 ms for the inner-square points and 8.2 to 9.3 ms for the outer-square points. In addition, leEPSC latency fluctuated largely on a trial-to-trial basis with outer-square stimulation (numbers 10, 12, 14, and 16; Fig. 4*C3*). Therefore, it is likely that the responses evoked by illumination of the outer-square sites resulted through polysynaptic activation of CeC neurons. The precise location and identity of these excitatory neurons secondary to the LPB inputs remain elusive. As a whole, the short and invariant latency responses with a larger amplitude were evoked in a manner dependent on the location and distance of the neuron being recorded from the illumination. Such monosynaptic-like responses were stably evoked when the illumination was within 500 μm from the soma of the recorded neurons, which probably corresponds to the spatial coverage of the light without ignorable peripheral decay using the ×40 objective. In addition, the negligibly small response or lack of a response to remote-site illumination (e.g., stimulation at number 15 in Fig. 4*A4*) strongly supports the notion that leEPSCs did not simply result from light emission but rather from the activation of ChR2-expressing fibers in specific sites in the slices. As shown in the next section, it is likely that transmitter release in response to illumination occurred mostly through activation of ChR2 at the terminals, and the contribution of axonal ChR2 might be smaller.

#### leEPSCs in CeC neurons are monosynaptic.

As demonstrated above (Fig. 2), LPB neurons expressing ChR2-EYFP responded to repeated light stimulation at 10 Hz with high fidelity. We then evaluated the effect of repeated light stimulation and postsynaptic responses, as well as action potential dependency using coronal slices. This was also the case with the postsynaptic responses. Blue light stimulation (duration 5 ms, 10 Hz) produced robust leEPSCs in response to each application (Fig. 5, *A1* and *A2*). Interestingly, the amplitude of leEPSCs to the second light pulse (i.e., light stimulation following a 100-ms interval) was much smaller than to the first light pulse (i.e., that following a 20-s interval), and the paired-pulse ratio (PPR) for these pairs of responses was 0.46 ± 0.04 (range, 0.30–0.70; *n* = 11). This is in contrast to the direct light-evoked responses observed in LPB neurons, in which the ratio of the second inward current amplitude evoked by light to the first one was 0.78 ± 0.02 (range, 0.75–0.84; *n* = 5; Fig. 2*B3*). In addition, the postsynaptic responses in CeC neurons showed some, but only a few, failures, especially at the second stimulation and thereafter; the failure rate for leEPSC_1_ was 0.9 ± 0.9% (range, 0–10%; *n* = 11) and that for leEPSC_2_ was 3.6 ± 2.4% (range, 0–20%; *n* = 11). These results suggest that the small PPR of the postsynaptic responses is mostly attributed to the high release probability of light-evoked release, and the contribution of ChR2 desensitization at a 100-ms interval is, if any, limited.

leEPSCs were abolished by the application of the sodium channel blocker TTX (1 μM; Fig. 5*A3*), suggesting that leEPSCs depend on the generation of action potentials by afferent fibers. This also suggests that the light-evoked currents through the ChR2 channels at the terminals of the LPB-CeC fibers, if they exist, do not depolarize the terminals to a sufficient level to activate voltage-dependent Ca^2+^ channels and trigger exocytosis. A potassium channel blocker, 4AP, has been widely used in such situations to cause sustained depolarization of the terminals so that the activation of ChR2 readily triggers synchronized transmitter release, if ChR2 channels are indeed present on the terminals (Cho et al. 2013; Felix-Ortiz et al. 2013; Hubner et al. 2014). As expected, the addition of 4AP (100 μM) made the leEPSCs reappear (Fig. 5*A4*). This suggests that blue light-evoked depolarization of the axon terminal of LPB-CeC fibers through ChR2 activation was large enough to trigger Ca^2+^ channel-dependent exocytosis, even in the absence of action potential generation under conditions where the terminals are depolarized additionally by 4AP. As the expression of ChR2 in the fiber terminal is strictly limited to those arising directly from transfected neurons, these results clearly indicate that leEPSCs were activated monosynaptically. This monosynaptic transmission was glutamatergic, because the further addition of Kyn (3 mM) abolished leEPSCs (Fig. 5*A5*). These results were confirmed in a total of 9–11 neurons, in which the area of leEPSCs (i.e., total charge transfer by leEPSCs) was evaluated and normalized to that before drug addition (Fig. 5*B*; “ACSF”). There was a statistically significant effect of the drugs on the leEPSC charge (*P* < 0.05, 1-way ANOVA with repeated measures, F[1.28,10.23] = 6.95 after Greenhouse-Geisser correction). The relative leEPSC_1_ charge was decreased significantly by the addition of TTX to −1.3 ± 2.3% (*P* < 0.005, Wilcoxon's signed-rank test; *n* = 11) and increased significantly to 98.8 ± 18.4% of the value with ACSF by the addition of 4AP (*P* < 0.02; *n* = 11). The further addition of Kyn significantly decreased the relative leEPSC charge to 0.9 ± 1.7% (*P* < 0.01; *n* = 9) of the value recorded with ACSF (Fig. 5*B*).

The light-evoked release of glutamate in ACSF in the absence of TTX and that in the presence of TTX and 4AP should involve distinct mechanisms. The former should be highly dependent on action potential generation at axons expressing ChR2, and its release would be triggered mostly by action potential-dependent activation of voltage-dependent Ca^2+^ channels after nerve conduction. In contrast, the latter would occur by the activation of voltage-dependent Ca^2+^ channels following ChR2 current-induced direct depolarization of the terminals, in addition to Ca^2+^ entry through ChR2 channels. This difference should affect release properties and timings. In accordance with this expectation, the PPR in the presence of TTX and 4AP (0.27 ± 0.05; *n* = 11; traces not shown) was significantly smaller (*P* < 0.005, paired *t*-test) than in the absence of TTX and 4AP shown above. The onset latency of leEPSCs in the presence of TTX and 4AP was significantly longer than that recorded in ACSF (before TTX + 4AP, 3.2 ± 0.1 ms; in the presence of TTX + 4AP, 5.3 ± 0.3 ms; *P* < 0.001, paired *t*-test; *n* = 11; Fig. 5*C1*). In addition, the SD of leEPSC latency (i.e., jitter) in the presence of TTX and 4AP was significantly larger than before pharmacological manipulation (before TTX + 4AP, 0.27 ± 0.06 ms; in their presence, 0.72 ± 0.21 ms; *P* < 0.05, paired *t*-test; *n* = 11; Fig. 5*C2*). We failed to find any specific difference between the data from neurons in the right and left amygdala. As a whole, these data further support the notion that light-evoked release occurred only at the terminals of fibers arising from neurons within and in the vicinity of the LPB.

#### Potent feed-forward inhibition following light-evoked activation of the LPB pathway.

The CeA is mostly composed of GABAergic inhibitory neurons, and this property defines the “gating” behavior of the intra-CeA network (Ehrlich et al. 2009). As excitatory afferent fibers pass in the very vicinity of the CeA in which GABAergic neurons are compactly and densely located, stimulation with a conventional electrode cannot avoid the risk of directly stimulating the GABAergic neurons in the CeA, especially with the strong stimulation intensity needed to activate afferent fibers fully. This risk has been avoided carefully in some previous studies by pharmacologically blocking GABA-mediated transmission (Cheng et al. 2011; Ikeda et al. 2007; Nakao et al. 2012), thereby preventing analysis of how these extrinsic excitatory inputs affect the network behavior of the CeA. An optogenetic approach can avoid this risk of directly stimulating nearby GABAergic neurons. In a neuron showing a robust inward current in response to blue light stimulation at −60 mV (Fig. 6, *A1* and *A2*), a very large, outward postsynaptic current was observed at a more depolarized holding potential (+15 mV; Fig. 6, *A1* and *A2*). At this depolarized potential, we confirmed that the *N*-methyl-d-aspartate receptor-mediated components evoked by blue light stimulation became minimum in a separate set of experiments (see below; Fig. 6, *D* and *E*). This outward postsynaptic current was abolished by TTX (Fig. 6*A3*), and unlike the inward current at −60 mV (Fig. 6*A4*), it did not reappear, even after the addition of 4AP (+15; Fig. 6*A4*), strongly suggesting that this outward component does not result from monosynaptic activation of LPB-CeC transmission. Figure 6*B* summarizes the effects of TTX, 4AP, and Kyn on the inward and outward charges recorded from the same set of neurons.

The timing of the outward current also differed from that of the inward current. As seen in the time-expanded version of the rising phase of these currents (Fig. 6*A2*), the outward current was more delayed, and its onset was more varied in time. The latency of this outward current was significantly longer than that of the inward current recorded in the same neurons (Fig. 6, *A2* and *C1*), and its SD was larger than that of the inward current (Fig. 6*C2*). These differences in latencies between the inward and outward currents are in agreement with recent studies on optogenetic monosynaptic and polysynaptic activation of EPSCs and inhibitory postsynaptic currents (IPSCs) in the hippocampus and basolateral amygdaloid nucleus (BLA) (Cho et al. 2013; Felix-Ortiz et al. 2013; Hubner et al. 2014), suggesting that the outward current was activated polysynaptically.

These outward postsynaptic currents observed at +15 mV were still observed in the presence of 50 μM AP5, a selective *N*-methyl-d-aspartate receptor antagonist (Fig. 6, *D* and *E*), but were abolished by 10 μM Bic. AP5 did not significantly change the total charge of this outward synaptic current (in the presence of AP5, 92.4 ± 14.5% of that before AP5; *n* = 9). Bic drastically reduced the amplitude of this current to 6.0 ± 1.7% of that in the presence of AP5 and to 5.1 ± 1.4% of that before AP5 (*P* < 0.01, Wilcoxon's signed-rank test; *n* = 9; Fig. 6*E*). These results confirm that GABA-mediated IPSCs account for the majority of these outward postsynaptic components. Thus it is highly likely that leEPSCs were followed by polysynaptic IPSCs arising from the action potential of GABAergic neurons, resulting from the light-evoked depolarization of these neurons.

Interestingly, we found a significant correlation between the outward and inward charges from the same neurons recorded at +15 mV and −60 mV, respectively (*r* = −0.730, Pearson's correlation coefficient, *P* < 0.001; *n* = 19; Fig. 6*F*). It is unlikely that this parallel relationship resulted simply from differences in the amount or efficacy of ChR2 transfection and expression between rats and preparations, because large or small inward and outward currents could be recorded from neurons from the same side of the same rats (Fig. 6*F*). Rather, this high correlation might indicate that neurons receiving a larger number of LPB-originating inputs also receive polysynaptic inputs from a larger number of local neurons. We failed to find any specific difference between the data from neurons in the right and left amygdala.

Altogether, optogenetic stimulation revealed that the inputs from neurons within and in the vicinity of the LPB not only directly excite CeC neurons but also activate feed-forward inhibition.

#### Selective activation of LPB pathways in horizontal slices and postsynaptic currents in CeM neurons.

All electrophysiological studies, to date, that analyzed LPB-CeC synaptic transmission used coronal (transverse) slices that allow visual identification of this pathway [e.g., see Fig. 9*B4* in Sarhan et al. (2005)] and exact placement of the electrode in the slice (Cheng et al. 2011; Delaney et al. 2007; Ikeda et al. 2007; Nakao et al. 2012; Neugebauer et al. 2003; Watabe et al. 2013). In contrast, horizontal slices of the amygdala have been used preferentially in many studies to understand the intranucleus connections from the CeC/CeL to the CeM, because of the improved preservation of connecting fibers between these distinct subnuclei (Sarhan et al. 2005; Viviani et al. 2011). However, because of the difficulty in identifying the dorso-ventral and medio-lateral projection trajectories of LPB-CeC fibers in horizontal slices, it has been practically impossible to stimulate this pathway in horizontal slices, despite their potential advantages in analyzing the network consequences of LPB inputs. Therefore, we examined whether the selective optogenetic activation of fibers arising from the LPB and adjacent structures would allow observation of the responses of CeM neurons to LPB inputs using horizontal slices. The EYFP-positive fibers were densely located at the most lateral part of the CeA, adjacent to the anterior part of the BLA or amygdalostriatal transition area at a slightly rostral level of the commissural stria terminalis (cst; Fig. 7*A3*). In contrast, the EYFP-positive fibers were much less dense in the CeM (Fig. 7, *A3* and *B*) at two different dorso-ventral levels (Fig. 7, *A2, a and b*–*A4*, *a* and *b*), in agreement with a previous report showing that LPB neurons have limited projections to the CeM [Sarhan et al. (2005), but see also D'Hanis et al. (2007)]. We recorded 10 neurons from the CeM in 6 slices from 4 rats expressing ChR2-EYFP (Fig. 7*A4*). Despite this sparser presence of EYFP-positive fibers, light illumination of CeM neurons triggered robust light-evoked responses with inward (recorded at −60 mV) and outward (+15 mV) postsynaptic currents, both of which were composed of multiple peaks appearing repeatedly for hundreds of seconds after light illumination in the majority of CeM neurons recorded (Fig. 7, *C1* and *C2*). In particular, these light-evoked responses were characterized by a relatively larger incidence and larger amplitude of putative IPSCs (Fig. 7*C2*; see Fig. 6*A2* for an example of a CeC neuron recorded in a coronal slice). Interestingly, such large and repeated outward current responses were observed, even in neurons showing small, inward postsynaptic responses. The ratio of the outward-to-inward charges of CeM neurons recorded in horizontal slices was significantly larger than that of CeC neurons recorded in coronal slices (CeM neurons, 9.22 ± 4.22, *n* = 10; CeC neurons, 2.40 ± 0.69, *n* = 6; *P* < 0.05, Mann-Whitney *U*-test; Fig. 7*D*; the data for the CeC neurons in coronal slices were from the same recordings as used in Fig. 6*B*). There was no right-left difference between three and seven neurons from the right and left CeM, respectively, for all of these measured values (*P* > 0.921, Mann-Whitney *U*-test). These responses were evoked by illumination centered on the CeM; however, such a response pattern remained essentially unchanged, even with CeC-centered illumination. This observation is in a good accordance with the results shown in Fig. 4, indicating that the effect of illumination was relatively insensitive to a shift in the illumination center by ∼500 μm (e.g., Fig. 7, *A3* and *A4*). This is the first study, to our knowledge, to record the responses of CeM neurons to parabrachial inputs in slice preparations with the use of optogenetics. As almost all CeC neurons responded to light illumination with a stable, monosynaptic excitatory response, followed by simple feed-forward inhibition (e.g., Fig. 6, *A* and *D*), the frequent and larger inhibitory inputs and relatively small, direct excitatory input in CeM neurons following light stimulation would suggest that these neurons integrate the activities of mostly GABAergic CeC (and presumably CeL) neurons, forming synapses with CeM neurons after parabrachial inputs.

#### Synaptic potentiation in CeC neurons in an inflammatory pain model, as revealed by light-evoked selective activation of LPB fibers.

It is widely acknowledged that cerebral mechanisms underlying “acute” and “chronic” pain are partly shared but largely separate (Baliki and Apkarian 2015). In particular, brain plasticity is an essential factor of the pain-chronification process. As such, the receipt of nociceptive information and generation of synaptic potentiation in response to sustained nociceptive or inflammatory information are distinct aspects of pain networks. In this context, the CeA not only receives nociceptive inputs but is also now established as a site of plastic changes during persistent pain (Carrasquillo and Gereau 2007; Hashmi et al. 2013; Neugebauer et al. 2004). To examine whether the monosynaptic inputs from the LPB and surrounding structures to CeC neurons evoked by blue light undergo nociception-associated plasticity, we investigated the effects of inflammatory pain on the light-evoked synaptic transmission in the CeC. For this purpose, we used the “latent postformalin” model, because this model is characterized by plastic changes in the CeA and hypersensitivity in a manner dependent on ERK phosphorylation at 6–24 h postinjection after the disappearance of the initial nocifensive behaviors (Adedoyin et al. 2010; Carrasquillo and Gereau 2007), in expectation that this latent state would represent a consolidating phase with a shift toward the chronic pain state, as suggested by various latent (>24 h) molecular changes in the spinal cord (Allen et al. 2003; Tan et al. 2012). Following injection of 5% formalin or saline into the left hindpaw, we observed and evaluated nocifensive behavior (licking of the injected paw) for 50 min. Except for the transition period (10-20 min postinjection) from the early to late phase responses, licking time in the formalin-treated rats was significantly longer than in the saline-treated rats (*P* < 0.01, Mann-Whitney *U*-test; Fig. 8*A*). At 24 h after injection, we made 25 coronal and 4 horizontal brain slices from 10 formalin- and 6 saline-treated rats and recorded leEPSCs at −60 mV in the presence of picrotoxin (Fig. 8*B*). All recordings for this series of experiments were made from the right CeC, which is predominantly activated by formalin injection (Carrasquillo and Gereau 2007). We recorded multiple neurons from single rats to confirm that the difference in leEPSC amplitude did not simply depend on transfection efficacy or the expression level of ChR2 in distinct preparations (Fig. 8*B* shows distinct neurons recorded in slices from single rats). In fact, we recorded neurons showing diverse amplitude ranges even from the same preparations. Contrary to our expectations, we failed to find a significant difference in leEPSC amplitude between the formalin- and saline-treated groups when data from all neurons in each group were pooled (*P* = 0.269 for all neurons between the saline- and formalin-treated groups, Mann-Whitney *U*-test; *n* = 41 and 57, respectively). However, as shown in Fig. 8*B*, the neurons from formalin-treated rats showed occasional large-amplitude leEPSCs (e.g., >250 pA). This suggests that despite robust direct postsynaptic inputs in all of these neurons, the capability to induce synaptic potentiation is a separate characteristic inherent to each neuron, as CeA neurons in rats as well as in mice are not a homogeneous population of neurons but rather, an assembly of neurons involved in various functions with distinct synaptic connectivity [Amano et al. (2012) and Chieng et al. (2006) for rats; Haubensak et al. (2010) for mice].

Therefore, we postulated that the formalin-induced synaptic potentiation of monosynaptic LPB-CeC connections would depend on the characteristics of postsynaptic neurons. Unfortunately, unlike in mice, the relationship between CeA neuron function and expression of marker proteins, such as somatostatin (SOM) (Li et al. 2013) and PKCδ (Haubensak et al. 2010), remains only poorly identified in rats, and the necessary molecular tools, such as animals with recombinase expression under specific molecular drivers, such as PKCδ-cre or SOM-cre mice (Haubensak et al. 2010; Li et al. 2013), are not yet available in rats. Therefore, as an alternative “second-best” approach, we compared leEPSC amplitude between cells with different firing properties (Amano et al. 2012; Dumont et al. 2002), which has been suggested to show a modest dependency on the functional role of neurons in rats (Amano et al. 2012). All three types of neurons—LTB, RS, and LF—showed robust leEPSCs (Fig. 8, *C1* and *C2*), indicating that CeC neurons receive monosynaptic inputs from the LPB and surrounding structures, regardless of neuronal type. There were differences, however, in leEPSC amplitude among different neuron types and between the saline- and formalin-treated groups (Fig. 8, *C3* and *D*). Figure 8*D* indicates a cumulative probability plot showing the distributions of leEPSC amplitude in three different types of neurons and in formalin- and saline-treated rats. The distribution was significantly different between the formalin- and saline-treated groups only in LF neurons (*P* < 0.05, Kolmogorov-Smirnov test), indicating that the distribution of leEPSC amplitude in LF neurons changes toward more frequent, larger-amplitude responses to LPB inputs. A similar conclusion could be drawn from nonparametric comparisons between distinct groups (Fig. 8*E*). Although all neurons with distinct firing types showed robust leEPSCs, the amplitude in LTB neurons was significantly smaller than in RS neurons in the saline-treated animals and also significantly smaller than in both RS and LF neurons in the formalin-treated rats (*P* < 0.01 for LF vs. LTB, and *P* < 0.05 for RS vs. LTB, Mann-Whitney *U*-test with Bonferroni post hoc compensation of *P* values; Fig. 8*E*). There was no significant difference in the portion of distinct cell types observed in the present recording conditions between saline- and formalin-treated rats (*P* = 0.433, χ^2^ test; Fig. 8*F*). In addition, there was no specific location of neurons that showed a specific firing pattern and also leEPSC responses with larger amplitude in saline- and formalin-treated animals (Fig. 8*G*), except that neurons with larger leEPSC amplitudes in formalin-treated rats were found frequently at a level slightly dorsal to the cst passage in the coronal plane (Fig. 8*G*; −2.04 in the coronal slice) and at almost the same rostrocaudal level as the cst passage in horizontal slices (Fig. 8*G*; −8.10 in the horizontal slices).

## DISCUSSION

The CeC is a subnucleus of the amygdala that receives nociceptive information (Bernard et al. 1992; Neugebauer and Li 2002). Previous studies indicated the following: *1*) direct axonal projections from the LPB to CeC, as revealed by anterograde tracing (Dong et al. 2010; Sarhan et al. 2005); *2*) evoked firing in response to LPB stimulation in anesthetized rats (Neugebauer and Li 2003); and *3*) glutamatergic postsynaptic responses recorded in CeC neurons in response to light stimulation after ChR2 expression in neurons with CGRP expression (Carter et al. 2013). These lines of evidence suggested that the LPB sends excitatory projections to the CeC. Here, with the use of projection-specific optogenetic activation of the LPB-CeC pathway, we have found the following: *1*) the LPB sends direct excitatory projections that form monosynaptic glutamatergic synapses with CeC neurons; *2*) such direct inputs could be recorded from a large majority of CeC neurons regardless of their firing pattern; *3*) excitatory postsynaptic responses in CeC neurons induced by light-evoked excitation of these fibers are followed by a large component of GABA receptor-mediated postsynaptic components; *4*) light-evoked excitation of these pathway results in inhibitory-predominant integrated and sustained responses in CeM neurons; and *5*) LPB-CeC synaptic transmission activated by optogenetic excitation shows robust potentiation in a persistent inflammatory pain model. Each of these findings is discussed below.

### 

#### Monosynaptic projections from the LPB and surrounding structures to the CeC.

Neugebauer and Li (2002) were the first to demonstrate the unitary responses of CeC neurons evoked by orthodromic stimulation with an electrode inserted in the LPB, suggesting that the anatomically identified projections from regions around the LPB to the CeC are excitatory (Gaurieau and Bernard 2002; Jasmin et al. 1997). In many previous studies using slice preparations, postsynaptic responses in CeC neurons were evoked by electrical stimulation of their afferent fibers with electrodes placed on putative regions medio-dorsal to the CeM, with the assumption that the fibers arising from the LPB were stimulated (Ikeda et al. 2007; Neugebauer et al. 2003; Watabe et al. 2013). Although these studies described that the placement of electrodes was made with special care (Ji et al. 2015; Watabe et al. 2013), there have always been questions as to the specificity of such stimulation methods, such as risk of stimulating other afferents converging on the CeC (Sah et al. 2003), especially at strong stimulation intensities, and risk of stimulating only a subset of projection fibers because of the diverse entrance routes of the LPB-CeC projections, as demonstrated by single fiber tracing [e.g., see Fig. 9*A* in Sarhan et al. (2005)]. Indeed, in the widely used “input-output” analysis of this synapse, that is, measurement of electrically evoked EPSC amplitude in response to increasing stimulation intensity (Bird et al. 2005; Ikeda et al. 2007), EPSC amplitude rises as stimulating intensity is increased, even at a strong intensity. This suggests that not all of the fibers are recruited, even with strong stimulation, and/or that increased stimulation intensity recruited additional fibers other than those with an origin in the LPB. This is in marked contrast to the present optogenetic stimulation in which leEPSC amplitude was insensitive to light intensity and duration above a certain low threshold. As AAVs do not trans-synaptically infect postsynaptic neurons regardless of their serotype, it is highly convincing that a fixed number of fibers arising from the LPB and surrounding structures made direct contact with CeC neurons (Betley and Sternson 2011; Luo et al. 2008). On the basis of this observation, it is likely that the present approach succeeded in activating almost the full set of fibers arising from the virally infected neurons within and around the LPB projecting to the CeC.

In this study, we used projection-specific optogenetic activation of the fibers putatively arising from the LPB. Although we carefully injected the AAV vector and rejected cases with out-of-place injections, the possibility of ChR2 expression in neighboring structures cannot be ruled out. In such cases, LED illumination should also have activated these fibers and terminals in the CeA. The immunohistochemical visualization of CGRP-expressing cells in the EYFP-expressing slices suggested that a subset of CGRP-positive neurons in and around the LPB would be a part of the neurons projecting to the CeC (D'Hanis et al. 2007). In addition to the LPB, a subset of neurons in the KF also projects to the CeA (Bianchi et al. 1998). Indeed, we observed faint but clear expression of EYFP at the level of the KF (immediately ventral to the LPB) in some preparations (e.g., Fig. 1*A3*). In contrast to the LPB and KF, projections to the CeA from the medial parabrachial nucleus (MPB) and dorsal LPB are scarce (Bianchi et al. 1998; Li et al. 2006). As KF neurons express vesicular glutamate transporters (Yokota et al. 2011), it is possible that the leEPSCs that we recorded might, in part, contain responses to fibers of KF origin. However, it has been shown that the KF receives nociception-related inputs mostly from trigeminal- and vagus-nerve innervated regions (Hermanson and Blomqvist 1997), and formalin injection to the hind limb, unlike that into the lip, results in the appearance of only a limited number of FOS-positive neurons in the KF. This suggests that the synaptic potentiation in the CeC in response to hind-limb formalin injection observed in this study would largely reflect activation of the LPB-CeC pathway.

#### CeC neurons receiving LPB and surrounding regions inputs.

One of the interesting findings of this study is that in slices with clear EYFP expression in the CeC, neurons without light-evoked responses were only recorded rarely. This suggests that most CeC neurons receive direct excitatory inputs from the LPB and surrounding structures. Recent studies on fear/threat learning have identified several distinct cell types in the CeA with different roles. For example, neurons in the CeC and CeL (collectively called “CeL” by researchers using conventional nomenclature) of the mice are divided into CeL-On and CeL-Off neurons; the former receive excitatory auditory conditional information for fear/threat learning and inhibit PKCδ-expressing CeL-Off neurons, which in turn inhibit CeM neurons. However, at least for the CeC neurons in the rat, the input from the LPB consistently exerted excitatory, and then followed by feed-forward inhibitory, influences. It is thus speculated that LPB inputs have a larger global influence on regulating CeA network excitability. Recently published behavioral studies, indicating that optogenetic activation of a subset of these LPB-CeC fibers by genetic (using CGRP expression in the LPB) (Han et al. 2015)- or projection (with similar virus transfection to that used in the present study; a report from our group)-dependent (Sato et al. 2015) ChR2 expression has a potent pro-learning effect in auditory fear/threat learning, support the notion that this pathway alone sends information sufficient to trigger the whole CeA activity that underlies potent adaptive memory and network reorganization. Recently, several molecular markers have been identified in the mouse CeA, such as PKCδ (Haubensak et al. 2010) and SOM (Li et al. 2013), and have been shown to be related to the specific roles of CeL (including CeC) and CeM neurons and proposed to underlie specific functions, especially in fear/threat learning (Ehrlich et al. 2009; Wolff et al. 2014). Although information for these molecules is limited in rats, and it is suggested that their role would be different from rats (Amano et al. 2012), it would be an important future subject to reveal the relationship between different neuronal types and their role in the nociception-emotion link.

#### Persistent acute inflammatory pain induces synaptic potentiation.

The most intriguing finding was the light-evoked synaptic potentiation of LPB-CeC synapses in the formalin-induced inflammatory pain model. This is the first demonstration that optogenetically activated monosynaptic excitatory LPB-CeC transmission undergoes pain-related plasticity. In various types of semi-acute to chronic pain models, such LPB-CeC, synaptic potentiation has been demonstrated using electrical stimulation of putative afferent pathways in slices (Adedoyin et al. 2010; Han and Neugebauer 2004; Ikeda et al. 2007; Nakao et al. 2012; Neugebauer et al. 2003; Ochiai et al. 2012). However, it has always remained a possibility that such stimulation also excites fibers other than those from the LPB, especially at a strong stimulation intensity. Indeed, a striking difference between our light-evoked stimulation and conventional electrical stimulation is that the amplitude of leEPSCs reached a plateau at a relatively weak light intensity, unlike the input-output curves of electrical stimulation experiments [e.g., see Fig. 1 in Bird et al. (2005)]. This suggests that the number of LPB fibers making synapses with each CeC neuron is somewhat limited so that the blue light stimulation of slices used in this study could activate these fibers fully. Therefore, this is the first demonstration that the postsynaptic responses to such almost-full presynaptic activation are increased in the persistent pain model. As described above, it is possible that in addition to the LPB, ChR2 protein is expressed in tissues surrounding the LPB following virus vector injection, and some of these, such as the KF, might project glutamatergic axons to the CeC. However, unilateral hind-limb injection of formalin reportedly results in only limited expression of FOS in the KF, suggesting that the KF is not primarily activated in this inflammatory hind-limb pain model (Hermanson and Blomqvist 1997). It is thus likely that the synaptic potentiation observed in the CeC of this pain model would reflect mostly synaptic potentiation between LPB-CeA projections.

Interestingly, the present approach also demonstrated that the degree of synaptic potentiation depended on the firing type of the neurons. To date, the relationship between the firing pattern and functional role of amygdala neurons, particularly in rats, also remains only poorly addressed. The mechanism underlying such firing pattern-dependent plasticity remains undetermined. One possibility is that only the synapses between LPB fibers and LF neurons are equipped with the necessary machinery for activity-dependent plasticity. Another important aspect worth noting is that we analyzed leEPSCs only at 24 h after formalin injection, according to a report by Adedoyin et al. (2010). The finding that only LF neurons show significant changes might not be the same at different time points because of the dynamic changes in molecular expression patterns during the chronification of inflammatory pain (Allen et al. 2003; Tan et al. 2012). Such time-dependent changes in synaptic plasticity in distinct neuronal components of the CeA network would be an important subject of future studies. In addition, recent studies using a similar type of optogenetic activation in mice from our laboratory and another suggest that this pathway also plays a role as a route providing a “teaching”-aversive signal to the amygdala circuit underlying fear/threat learning (Han et al. 2015; Sato et al. 2015). As we have already demonstrated that the postsynaptic current evoked by electrical stimulation of the putative LPB-CeC pathway is enhanced following electrical shock-primed fear/threat learning (Watabe et al. 2013), it is also highly possible that the potentiation after formalin injection observed in this study would also affect associative memory against harmful events and lead to mood/affective complications.

In the present study, the postsynaptic currents were recorded at room temperature, which enabled long-term, stable recordings, even in the slices from aged rats (>9 wk old). It should be noted, however, that the properties of synaptic plasticity depend on the uptake of transporters around synapses, which depends on temperature (Asztely et al. 1997; Tsvetkov et al. 2004). Therefore, the characteristics of the synaptic transmission, particularly those of pain-related potentiation, described in the present study, will not exactly be the same at the conditions more relevant to the physiological situations. The final consequences of the balance between the monosynaptic excitation and feed-forward inhibition in the CeC neurons, as well as the excitation/inhibition balance in the CeM neurons, as found in this study during the course of pain chronification at physiological environments, would be an important subject for future studies in understanding the role of the CeA network in a potentiated nociception–emotion link and CeA-mediated regulation of the nociceptive behaviors (Carrasquillo and Gereau 2007).

#### Potent feed-forward inhibition of LPB inputs.

Another important, novel finding of this study is the potent inhibitory component mediated by GABA_A_ receptors that immediately follows the leEPSC. This is reminiscent of a similar feed-forward inhibition reported recently in the bed nucleus of the stria terminalis (BNST) after light activation of ChR2, also in LPB-origin fibers (Flavin et al. 2014). This finding provides a novel insight into the functional significance of the nociception-related inputs arising from the LPB in CeC network signaling. First, the impact of these inputs might depend on the interval of afferent fiber discharges. For example, there was an ∼7-ms difference, on average, in poststimulus latency (Fig. 6*C1*) and an ∼10-ms difference in peak timing (Fig. 6*A2*) between the inward (leEPSC) and outward (light-evoked polysynaptic IPSC) components. This means that an action potential of an LPB fiber, arriving at 7–10 ms after the previous one, cannot generate an EPSC as large as the previous one in CeC neurons, because it coincides with the peak of this feed-forward IPSC, which shunts the membrane at this moment. Second, because the duration of IPSCs is generally longer than that of EPSCs (Fig. 6*A2*), such inhibitory effects would accumulate, especially with repeated high-frequency discharges of LPB-CeC fibers. It is thus speculated that the role of nociceptive input in the regulation of CeA activity is not simply an excitatory one but rather, modified depending on the frequency of repeated inputs and also by the balance between direct excitation and polysynaptic inhibition.

The origin of these inhibitory components was not explored in the present study. Its identification is an important research subject to understand the network behavior of the inhibitory circuits of the CeA (Ehrlich et al. 2009; Haubensak et al. 2010). Possible candidates include the following: *1*) GABAergic neurons in the CeA and/or CeL (Delaney et al. 2007; Dong et al. 2010; Sarhan et al. 2005); *2*) those in the BNST (Flavin et al. 2014; Sarhan et al. 2005); and *3*) those in the intercalated cell mass (Ehrlich et al. 2009). As the intercalated cell mass did not show detectable EYFP expression, even in slices with dense EYFP signals in the CeC (see Figs. 3*A3* and 4*A3*), and as the BNST neurons were not included in the slices used, the involvement of these structures in generating feed-forward inhibition would be limited. The location of GABAergic neurons that receive monosynaptic LPB inputs and target CeC neurons remains to be identified in future studies using more elaborate techniques to visualize neuronal excitation in the entire network. As most CeC neurons showed leEPSCs in the present study, such connections would enable the firing of other CeC neurons in response to light stimulation and give rise to delayed polysynaptic IPSCs. In this context, it could be argued that this is not simply a case of conventional feed-forward inhibition but rather, a case of afferent sensory information-dependent modulation of the efficacy of network gating (Ehrlich et al. 2009; Veinante et al. 2013).

In addition, the present study is the first, to our knowledge, to record the responses of CeM neurons to LPB fiber activation with the optogenetic approach in horizontal slices, which has been otherwise impossible. The results indicated that CeM neurons show more complex and integrated patterns, predominantly composed of sustained, inhibitory inputs after single LPB fiber activation, than the simple feed-forward inhibition observed in CeC neurons in coronal slices. Although it remains to be clarified whether CeC neurons also show such predominant inhibitory synaptic inputs in the same horizontal slices, this observation is interesting and important, because the CeM provides the output from the CeA to structures underlying various functions regulating emotion and pain behaviors (Viviani et al. 2011). In particular, this finding is in contrast to the effect of excitatory auditory inputs arising from the BLA to the CeM, which are predominantly excitatory (Duvarci et al. 2011). In this regard, whether the same sets of CeC, CeL, and CeM neurons are essentially involved in pain-related emotion and fear/threat learning, as well as other mood, affective, and cognitive disorders, is an interesting and important hypothesis to be tested. The physiological significance of such inhibitory connections in the CeC and CeM, in response to presumably nociception-related inputs, remains to be identified in future studies, in which such optogenetic stimulation of specific afferent fibers is an obligatory approach. Thus this approach would be a promising tool to understand the role of nociceptive inputs and their chronic pain-associated plasticity in network processing from the CeC and then to the CeM and the outputs from the CeA of the amygdala to various brain sites involved in pain- and fear-associated behaviors and autonomic responses.

## GRANTS

Support for this work was provided by the following: a Grant-in-Aid for Exploratory Research from the Ministry of Education, Culture, Sports, Science, and Technology (MEXT; No. 23650208; to F. Kato), MEXT-Supported Program for the Strategic Research Foundation at Private Universities (S1311009; to F. Kato), a Grant-in-Aid for Scientific Research (B; 25293136; to F. Kato), the Strategic Research Program for Brain Sciences (to A. M. Watabe and F. Kato), the Japan Science and Technology Agency, PRESTO (to A. M. Watabe), a Grant-in-Aid for Scientific Research on Innovative Areas “Memory dynamism” (26115523; to A. M. Watabe), a Grant-in-Aid for Scientific Research (C; to A. M. Watabe), a Grant-in-Aid for Young Scientists (B; to Y. Takahashi), a Grant-in-Aid for Japan Society for the Promotion of Science (JSPS) Fellows (to Y. K. Sugimura), and a grant from the Research Foundation for Opto-Science and Technology (to F. Kato).

## DISCLOSURES

The authors declare no conflict of interest regarding the contents of this study.

## AUTHOR CONTRIBUTIONS

Y.K.S., Y.T., A.M.W., and F.K. conception and design of research; Y.K.S. performed experiments; Y.K.S. and F.K. analyzed data; Y.K.S. and F.K. interpreted results of experiments; Y.K.S. and F.K. prepared figures; Y.K.S. and F.K. drafted manuscript; Y.K.S., Y.T., A.M.W., and F.K. edited and revised manuscript; Y.K.S., Y.T., A.M.W., and F.K. approved final version of manuscript.
